# Association of Sleep Duration With Atrial Fibrillation and Heart Failure: A Mendelian Randomization Analysis

**DOI:** 10.3389/fgene.2021.583658

**Published:** 2021-02-24

**Authors:** Jianqiang Zhao, Fangkun Yang, Chengui Zhuo, Qiyue Wang, Zihao Qu, Qiqi Wang, Liangrong Zheng

**Affiliations:** ^1^Department of Cardiology and Atrial Fibrillation Center, The First Affiliated Hospital, School of Medicine, Zhejiang University, Hangzhou, China; ^2^Department of Cardiology, The Second Affiliated Hospital, School of Medicine, Zhejiang University, Hangzhou, China; ^3^Department of Cardiology, Taizhou Hospital of Zhejiang Province, Wenzhou Medical University, Taizhou, China; ^4^School of Medicine, Zhejiang University City College, Hangzhou, China; ^5^Department of Orthopedic Surgery, The Second Affiliated Hospital, School of Medicine, Zhejiang University, Hangzhou, China

**Keywords:** mendelian randomization, sleep duration, disturbed sleep, atrial fibrillation, heart failure

## Abstract

Both short (<7 h per night) and long (≥9 h per night) sleep durations are related to atrial fibrillation (AF) and heart failure (HF), but their causality has not been confirmed. We applied Mendelian randomization (MR) approaches to estimate the causal association between genetically determined sleep duration and the risk of AF and HF. We performed two-sample MR analysis to obtain the effect of sleep duration on AF and HF. Instrumental variables were constructed using genetic variants known to be associated with continuous sleep duration, short sleep duration, and long sleep duration. MR estimates of the effect of sleep duration on AF and HF were derived based on two large meta-analyses of genome-wide association studies. The pooled MR estimate demonstrated a significant protective effect of continuous sleep duration on HF [odds ratio (OR) = 0.765, 95% confidence interval (CI) = 0.675–0.867; *P* = 2.64 × 10^–5^] and a suggestive inverse association of continuous sleep duration with AF (OR = 0.893, 95% CI = 0.804–0.991; *P* = 0.034). In addition, the results showed a suggestive detrimental effect of short sleep duration on the risk of AF (OR = 1.108, 95% CI = 1.017–1.207; *P* = 0.019) and HF (OR = 1.136, 95% CI = 1.025–1.258; *P* = 0.015). Conversely, there is no significant evidence for the causal protective effect of long sleep duration on AF (OR = 0.956, *P* = 0.410) and HF (OR = 0.921, *P* = 0.202). This MR study indicated that genetically determined continuous sleep duration has a significant protective effect on HF and a suggestive inverse association with AF. Short sleep duration is positively associated with the risk of AF and HF. Nevertheless, there is no significant evidence for the causal protective effect of long sleep duration on AF and HF. Larger intervention studies are required to confirm the effectiveness of improving sleep on reducing the incidence of AF and HF.

## Introduction

Disturbed sleep is prevalent in a modern society (Akerstedt and Nilsson, 2003). It is well known that poor sleep quality is associated with increased risk of various health problems, such as obesity (Wu et al., 2014), diabetes mellitus (Cappuccio et al., 2010), hypertension (Gangwisch et al., 2006), cardiovascular diseases (Covassin and Singh, 2016; Li et al., 2020), renal disease (Geng et al., 2019), dementia (Fan et al., 2019), metabolic syndrome (Song et al., 2016), and other chronic health conditions (Stenholm et al., 2019). Several observational studies and meta-analysis studies have suggested that both short (<7 h per night) and long (≥9 h per night) sleep durations are related to atrial fibrillation (AF) (Song et al., 2017; Genuardi et al., 2019; Morovatdar et al., 2019) and heart failure (HF) (Aggarwal et al., 2013; Javaheri et al., 2016; Wannamethee et al., 2016). Conversely, the causality of the association between sleep duration and AF and HF has not been confirmed. Therefore, it is necessary to elucidate the role of sleep duration in AF and HF to clarify whether better sleep habits could reduce the risk of AF and HF.

Because of difficulty in disentangling causal from spurious effects due to confounding and reverse causation, randomized clinical trials investigating the association between sleep duration and AF and HF would be difficult. In recent years, Mendelian randomization (MR) approach has been widely used in estimating the causal effect of clinical factors with diseases. Based on the meta-analysis of genome-wide association studies (GWASs), MR approach utilizes genetic variants, typically the single-nucleotide polymorphisms (SNPs), to analyze the causality between exposures and outcomes. Random assignment of an individual’s genetic variants at conception is employed as instrumental variables; MR analysis could largely overcome the limitations of environmental confounders (Smith and Ebrahim, 2003; Sheehan et al., 2008). With the application of MR approach, associations between sleep duration and coronary artery disease (CAD), myocardial infarction (MI), and stroke have been demonstrated. Daghlas et al. (2019) supported short sleep duration as a potentially causal risk factor for CAD and MI, whereas Zhuang et al. (2020) found no significant association between sleep duration with stroke. The aims of the current study were to estimate the effects of genetically determined sleep duration on the risk of AF and HF with MR analysis. For completeness, we also reported results for the associations between sleep duration and ischemic stroke (IS) and its subtypes.

## Methods

**FIGURE 1:**
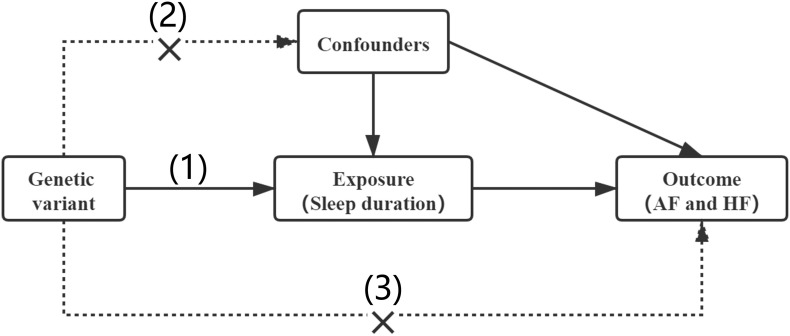
Assumptions of a Mendelian randomization analysis. Three core assumptions: (1) genetic variants must be strongly associated with the exposure (sleep duration); (2) genetic variants should be independent of any other confounders; (3) genetic variants influence risk of the outcome (AF and HF) only through the exposure.

In our MR analysis, the genetic variant qualified as a valid instrument for causal inference must satisfy the following three essential assumptions: (1) genetic variants must be strongly associated with the exposure (sleep duration); (2) genetic variants should be independent of any other confounders; and (3) genetic variants influence risk of the outcome (AF and HF) only through the exposure (Figure 1).

### Genetic Association Estimates

All genetic variants reaching genome-wide significance (*P* < 5 × 10^–8^) were selected as instruments for the MR analysis. The corresponding linkage disequilibrium was tested on the LD-link website^1^ (European; *r*^2^ < 0.1). The instrumental variables of sleep duration were identified from the hitherto largest GWAS meta-analysis in individuals of European ancestry in the UK Biobank (Dashti et al., 2019), which have identified 78 SNPs associated with continuous sleep duration (*n* = 446,118), 27 SNPs associated with short sleep duration (<7 h; *n* = 106,192 cases/305,742 controls), and eight SNPs associated with long sleep duration (≥9 h; *n* = 34,184 cases/305,742 controls). Details of individual association estimates for sleep duration are illustrated in Supplementary Tables 1–3.

Genetic association data for AF were obtained from the latest meta-analysis of GWASs for AF, which included 1,030,836 European ancestry individuals from six contributing studies (60,620 with AF and 970,216 control), including the Atrial Fibrillation Genetics (AFGen) Consortium, the Nord-Trøndelag Health Study (HUNT), the Michigan Genomics Initiative (MGI), DiscovEHR, Collaborative analysis of Diagnostic criteria in Europe study (DECODE), and UK Biobank. AF was defined by electrocardiogram and *International Classification of Diseases* (*ICD*) codes for AF or flutter [*ICD-9*: 427.3, 427.31, or 427.32; *International Statistical Classification of Diseases and Related Health Problems* (*ICD-10*): I48] (Nielsen et al., 2018). For the effects of SNPs on HF, we included the summary statistics from the Heart Failure Molecular Epidemiology for Therapeutic Targets (HERMES) Consortium, which included 47,309 cases and 930,014 control subjects of European ancestry from 26 studies. HF was defined as the presence of self-reported HF/pulmonary edema or cardiomyopathy at any visit and *ICD* codes for HF (*ICD-10* or *ICD-9* billing code indicative of heart/ventricular failure or a cardiomyopathy of any cause) (Shah et al., 2020). Summary statistics for AF and HF were obtained from the UK Biobank Resource (361,194 participants of European ancestry, 167,020 men/194,174 women) to investigate the impact of sex difference on causal associations between sleep duration and these two diseases (Sudlow et al., 2015). Summary statistics data for IS and its subtypes were selected from a multiancestry GWASs of 29 studies by the MEGASTROKE consortium, and there were 60,341 cases of IS, 6,688 cases of large artery stroke, 9,006 cases of cardioembolic stroke, and 11,710 cases of small vessel stroke (Malik et al., 2018).

### Statistical Analysis

A two-sample MR method was used in the current study. The whole process of the MR is shown in the Supplementary Material. We used the Wald estimator to derive MR estimates of the effect of sleep duration on AF and HF, which is the ratio of the SNP-outcome genetic effect over the SNP-exposure genetic effect. The Delta method was used to account for possible measurement error in both the exposure and outcome association estimates (Brion et al., 2013; Thompson et al., 2016). The fixed-effects inverse variance-weighted (IVW) method was used to derive the final effect estimate for the main analyses. Estimates for the continuous sleep duration trait were scaled to hours by multiplying per-minute betas and SEs by 60. The bidirectional MR analyses were performed to test the bidirectional link between sleep duration with AF and HF. In addition, sensitivity analyses were performed using estimates for the effects of the variants on continuous sleep duration from the meta-analysis results [including 503,852 individuals from the UK Biobank, EArly Genetics and Lifecourse Epidemiology (EAGLE) and Cohorts for Heart and Aging Research in Genomic Epidemiology (CHARGE) consortium], weighted median (Bowden et al., 2016), and MR-Egger regression (Bowden et al., 2015) methods. Potential pleiotropy of the genetic variants, which would have influenced the outcome through pathways other than the exposure, was accounted by MR-Egger method. *cis*-MR analyses were performed using *cis*-acting SNPs (chromosome 3, 4, 11) that showed significant associations between sleep duration and AF and HF in the above analysis. The results of the present study are shown as odds ratio (OR) and 95% confidence interval (CI) per hour increase in continuous sleep duration and per doubling in the risk of short and long sleep durations. All statistical analyses were performed using R software (version 3.6.1) with the MR package, a Bonferroni-corrected level of significance of less than 0.008 (correcting for three exposures and two outcomes) was considered to indicate statistical significance. *P* values between 0.008 and 0.05 were regarded as suggestive evidence of associations.

## Results

The associations between instrumental variables for sleep duration and all the outcomes are demonstrated in Supplementary Tables 4–9 and details of studies and datasets used for analyses are displayed in Table 1. Genetic association estimates for the rs11602180, rs549961083, and rs5757675 were not available for HF. In addition, the *P* value of the association between two SNPs (rs1991556, rs17688916) and AF and two SNPs (rs11190970, rs17817288) and HF was lower than 1.00 × 10^–5^ (Supplementary Tables 4–6). Therefore, IVW analyses were performed on AF and HF after removing these SNPs from instrumental variables, respectively. Calculation of linkage disequilibrium of selected SNPs is shown in Supplementary Tables 10–12. The MR scatter plots and forest plots for each SNP are presented in Supplementary Figures 1–4. Results of *cis*-MR are presented in Supplementary Figure 5.

**TABLE 1:** Details of studies and datasets used for analyses.

**Exposures/Outcomes**	**Consortium**	**Ethnicity**	**Sample size**	**Cases**	**Controls**	**References**
Continuous sleep duration	UK Biobank	European	446,118	–	–	Dashti et al. (2019)
Short sleep duration	UK Biobank	European	411,934	106,192	305,742	
Long sleep duration	UK Biobank	European	339,926	34,184	305,742	
Atrial fibrillation	AFGen, HUNT, MGI, DECODE, and UK Biobank	European	1,030,836	60,620	970,216	Nielsen et al. (2018)
Heart failure	HERMES	European	977,323	47,309	930,014	Shah et al. (2020)

## Genetically Determined Sleep Duration With AF

**FIGURE 2:**
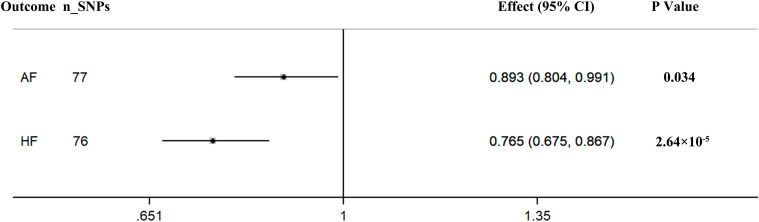
Causal associations between continuous sleep duration and AF and HF. The number of genetic variants, effects, 95% confidence intervals, and *P* values of associations are contained. n_SNPs, the number of SNPs used as instrumental variables; Effect, the combined causal effect; CI, confidence interval; *P* value, *P* value of the causal estimate.

**FIGURE 3:**
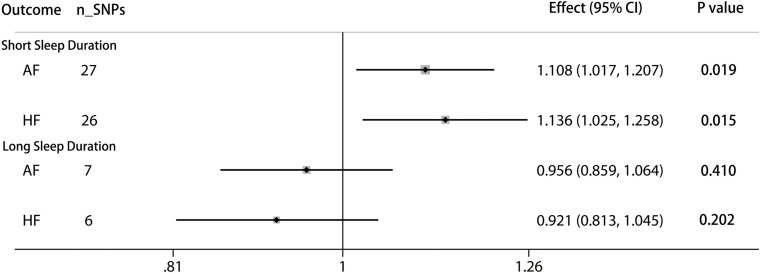
Causal associations between short/long sleep duration and AF and HF. The number of genetic variants, effects, 95% confidence intervals, and *P* values of associations are contained. n_SNPs, the number of SNPs used as instrumental variables; Effect, the combined causal effect; CI, confidence interval; *P* value, *P* value of the causal estimate.

The pooled MR estimate showed a suggestive inverse association of genetically predicted in continuous sleep duration with AF (Figure 2). For a 1-h increase in continuous sleep duration, the OR of AF was 0.893 (95% CI = 0.804–0.991, *P* = 0.034). Sensitivity IVW gained a similar result (OR = 0.864, 95% CI = 0.960–0.984; *P* = 0.027) (Supplementary Table 13). There was a suggestive evidence of a causal effect of short sleep duration on AF (OR = 1.108, per doubling in risk of short sleep duration; 95% CI = 1.107–1.207; *P* = 0.019) (Figure 3). Results from the weighted median method were similar to the IVW estimates (Supplementary Table 13). However, there was no association between genetically predicted long sleep duration and AF (OR = 0.956, per doubling in risk of long sleep duration; 95% CI = 0.859–1.064; *P* = 0.410) (Figure 3). The Egger intercept test did not show evidence of directional pleiotropy in the analyses (Supplementary Table 13). Sex-specific analyses showed suggestive causal effects of continuous sleep duration (OR = 0.989, 95% CI = 0.981–0.998; *P* = 0.019) and short sleep duration (OR = 1.008, 95% CI = 1.001–1.015; *P* = 0.031) on AF in men but not in women (Supplementary Table 13). The bidirectional MR analyses did not show a causal association between AF with sleep duration (Supplementary Tables 14–15, 18).

### Genetically Determined Sleep Duration With HF

According to the IVW analyses results, the OR for a 1-h increase in continuous sleep duration within HF was 0.765 (95% CI = 0.675–0.867; *P* = 2.64 × 10^–5^), suggesting a significant protective effect of continuous sleep duration on HF (Figure 2). In addition, MR remained significant when using summary statistics extracted from the meta-analysis results (OR = 0.780, 95% CI = 0.680–0.894; *P* = 3.70 × 10^–4^) (Supplementary Table 13). The MR analysis found a suggestive detrimental effect of short sleep duration on the risk of HF (OR = 1.136, per doubling in risk of short sleep duration; 95% CI = 1.025–1.258; *P* = 0.015). The same association was observed in sensitivity analyses, but the results were not statistically significant (Supplementary Table 13). However, there was no significant evidence for the causal protective effect of long sleep duration on HF (OR = 0.921, per doubling in risk of long sleep duration; 95% CI = 0.813–1.045; *P* = 0.202) (Figure 3). Sex-specific analyses indicated an obvious sex difference in the associations of continuous sleep duration and HF (OR = 0.995, 95% CI = 0.991–0.999; *P* = 0.021) (Supplementary Table 13). The bidirectional MR analyses did not show a causal association between HF with sleep duration (Supplementary Tables 16–18).

Genetically predicted sleep duration was not associated with IS and its subtypes (Supplementary Tables 19–22).

## Discussion

In the current MR study, we for the first time found the evidence that an increase in genetically determined continuous sleep duration has a causal protective effect on AF and HF. The MR analysis supported previously observed significant causal association of short sleep duration with risk of AF and HF. The protective effect of long sleep duration on AF and HF was observed in our study, but the results were not statistically significant. In addition, our study provided the very limited evidence on the sex-specific effects of sleep duration on AF and HF, which suggested a stronger effect for male than female. However, the bidirectional link between AF or HF and sleep duration was not confirmed in this MR analysis.

As a complex physiological process, sleep involves several different biological pathways, from neural cortical circuits to the heart (Saper et al., 2005). According to recommendations of the National Sleep Foundation, 7 to 9 h per night is the appropriate sleep duration for adults (Hirshkowitz et al., 2015). Previous studies suggested that alterations of physiological sleep are associated with cardiovascular diseases and vice versa (Aggarwal et al., 2013; Covassin and Singh, 2016; Bertisch et al., 2018; Deschênes et al., 2019).

The association between sleep duration and incident AF in the general population is mainly observed in different regions. In line with our findings, short sleep duration was reported to be positively associated with the incidence of AF in the particular Chinese and Japanese population (Han et al., 2017; Lee et al., 2017). In contrast, a recent meta-analysis study suggested an absence in association between AF and sleep duration (Chokesuwattanaskul et al., 2018), which was inconsistent with the results of our study. Multiple mechanisms are postulated to be responsible for AF induced by short sleep duration. Some studies suggested that short sleep duration is regarded as a risk factor for AF through autonomic dysregulation and structural remodeling (Xu et al., 2013; Deschênes et al., 2019). In addition, sleep deprivation has been shown to activate proinflammatory systems and oxidative stress (Kanagasabai and Ardern, 2015), which are considered as additional pathways predisposing to AF (Guo et al., 2012). Dysregulation of the hypothalamic–pituitary axis caused by insomnia can increase cortisol level and blunt heart rate variability (Bonnet and Arand, 1998). On the other hand, obstructive sleep apnea (OSA), a major cause of short sleep, is increasingly recognized as a risk for AF (Kwon et al., 2018). All these risk factors eventually contribute to the development of AF.

Epidemiological data show a higher prevalence of chronic insomnia in patients with HF than in the general population (Hayes et al., 2009). Besides, the American College of Cardiology/American Heart Association guidelines suggest that poor quality of sleep is often associated with worse compliance and outcomes in patients with HF (Riegel et al., 2009). Self-reported insomnia symptoms were found to predict increased risk of incident HF in a prospective cohort study (Ingelsson et al., 2007). However, short sleep duration is often related to but distinct from insomnia. Very few data are available on the effect of sleep duration on HF. Purported mechanisms between sleep duration and HF remains unclarified. Similarly, OSA (Gottlieb et al., 2010) and adverse cardiac remodeling (Javaheri et al., 2016) also have been considered to lead to increased risk of HF. Moreover, short sleep duration may cause less restorative sleep and impaired resetting of important reflexes leading to hypoxia and more severe cardiac dysfunction (Türoff et al., 2017). Furthermore, short sleep duration may reflect depression, which has been reported as an independent risk factor for incident HF (Cené et al., 2012). Anyway, the role of sleep duration on HF is only partially understood and needs to be further explored.

Furthermore, several loci that showed significant associations between sleep duration and AF and HF in this study have been found to contribute to the increased risk of cardiovascular diseases. Protein phosphatase 2 regulatory subunit B alpha (PPP2R3A, chromosome 3) was reported to be involved in HF and to play an important role in normal myocardium formation and efficient cardiac contractile function (Yang et al., 2016). Previous studies suggested that the BUD13 (chromosome 11) (Xu et al., 2018) and BANK1 (chromosome 4) (Hong et al., 2015) genes could affect blood lipid levels and increase cardiovascular risk. The *cis*-MR analyses revealed the causal protective effect of the BANK1 locus on AF (OR = 0.528, 95% CI = 0.322–0.867; *P* = 0.012) and HF (OR = 0.441, 95% CI = 0.247–0.785; *P* = 0.005), as well as the BUD13 locus on HF (OR = 0.541, 95% CI = 0.381–0.768; *P* = 0.021). However, there was no significant evidence for the protective effect of the PPP2R3A locus on AF or HF (Supplementary Figure 5).

In this MR study, we estimated the potential association between genetically predicted sleep duration on the risk of AF and HF. Based on the meta-analysis of GWAS datasets, the two-sample MR analysis was applied using SNPs to analyze the causality between exposures and outcomes. Random assignment of an individual’s genetic variants at conception is employed as instrumental variables; MR analysis could largely overcome the limitations of environmental confounders and provide high-quality evidence. In addition, we used the MR-Egger method to detect the pleiotropic effects in this study. In order to minimize the bias of the genetic variants’ frequencies, only European participants were included in our study.

## Limitations

Our study also had several limitations. Results of the IVW and weighted median analyses were contradictory, which might be caused by the potential differences in validity of all the SNPs. Second, only European individuals were included in this study. Further studies incorporating populations of different races are required for more conclusive results. Finally, the use of self-reported rather than objective sleep duration assessment (Matthews et al., 2018) and selection of relatively healthy participants into UKB, which might induce collider bias (Fry et al., 2017).

## Conclusion

This MR study indicated that genetically determined continuous sleep durations have a significant protective effect on HF and a suggestive inverse association with AF. Short sleep duration is positively associated with the risk of AF and HF. Nevertheless, there is no significant evidence for the causal protective effect of long sleep duration on AF and HF. Larger intervention studies are required to confirm the effectiveness of improving sleep on reducing the incidence of AF and HF.

## Data Availability Statement

The summary statistics of the UK Biobank and GWAS datasets used in this study are available on request provided there is a clear statement of purpose.

## Ethics Statement

All the studies used to compliment the current study were approved by relevant ethics committees. All participants involved provided a written informed consent.

## Author Contributions

JZ designed the study, contributed to the data analysis, and wrote the manuscript. FY and CZ contributed to the data analysis and data interpretation. QW and ZQ contributed to manuscript writing and revision of the manuscript. All authors read and approved the final draft of the manuscript.

## Conflict of Interest

The authors declare that the research was conducted in the absence of any commercial or financial relationships that could be construed as a potential conflict of interest.

## Footnotes

1 https://ldlink.nci.nih.gov/

## Abbreviations

AF: atrial fibrillation
HF: heart failure
MR: Mendelian randomization
GWAS: genome-wide association study
SNP: single-nucleotide polymorphism
IS: ischemic stroke
IVW: inverse variance-weighted
OR: odds ratio
CI: confidence interval
CAD: coronary artery disease
MI: myocardial infarction.
