# Supplementary material for: Association of Sleep Duration With Atrial Fibrillation and Heart Failure: A Mendelian Randomization Analysis
Source: Front Genet. 2021 Feb 24;12:583658. doi: 10.3389/fgene.2021.583658 (PMC7943616; doi:10.3389/fgene.2021.583658)

Supplementary Material

Table 1. Characteristics of SNPs for continuous sleep duration instrument from GWAS meta-analysis

| SNP | Chromosome position | Nearest Gene | Alleles  (E/A) | EAF | Info | F-statistic | Original-analysis | | | Meta-analysis | | |
| --- | --- | --- | --- | --- | --- | --- | --- | --- | --- | --- | --- | --- |
|  |  |  |  |  |  |  | Beta (min) | SE (min) | P value | Beta (min) | SE (min) | P value |
| rs915416 | 1:34731984 | CSMD2 | C/G | 0.290 | 1.00 | 68 | 1.156 | 0.150 | 9.9E-15 | 0.966 | 0.138 | 2.1E-12 |
| rs269054 | 1:57864304 | DAB1 | A/T | 0.422 | 1.00 | 41 | 0.819 | 0.138 | 2.1E-09 | 0.666 | 0.126 | 1.5E-07 |
| rs61796569 | 1:66476437 | PDE4B | T/C | 0.270 | 0.98 | 42 | 0.927 | 0.154 | 1.5E-09 | 0.828 | 0.144 | 4.5E-09 |
| rs12567114 | 1:98527951 | DPYD | A/G | 0.276 | 0.99 | 39 | 0.890 | 0.152 | 4.3E-09 | 0.846 | 0.138 | 2.1E-09 |
| rs11190970 | 10:103128332 | BTRC | G/A | 0.799 | 1.00 | 34 | 0.923 | 0.169 | 4.6E-08 | 0.864 | 0.162 | 6.5E-08 |
| rs7915425 | 10:125016501 | BUB3 | T/C | 0.175 | 1.00 | 47 | 1.144 | 0.179 | 2.0E-10 | 1.014 | 0.162 | 8.2E-10 |
| rs12246842 | 10:21830580 | MLLT10 | A/G | 0.460 | 1.00 | 40 | 0.804 | 0.136 | 3.9E-09 | 0.726 | 0.126 | 7.2E-09 |
| rs10761674 | 10:64618340 | EGR2 | C/T | 0.477 | 1.00 | 34 | 0.740 | 0.136 | 4.2E-08 | 0.696 | 0.126 | 2.5E-08 |
| rs1939455 | 11:101520886 | TRPC6 | G/T | 0.879 | 0.95 | 40 | 1.226 | 0.214 | 1.2E-08 | 1.050 | 0.198 | 1.7E-07 |
| rs7115226 | 11:113408518 | DRD2 | A/C | 0.074 | 0.98 | 43 | 1.594 | 0.261 | 1.7E-09 | 1.594 | 0.261 | 1.7E-09 |
| rs1263056 | 11:116576415 | BUD13 | A/G | 0.519 | 0.99 | 36 | 0.768 | 0.137 | 2.0E-08 | 0.768 | 0.126 | 1.3E-09 |
| rs7951019 | 11:118358027 | KMT2A | G/T | 0.032 | 0.96 | 38 | 2.213 | 0.391 | 1.2E-08 | 2.213 | 0.391 | 1.2E-08 |
| rs1057703 | 11:122830251 | BSX | G/T | 0.147 | 0.99 | 42 | 1.164 | 0.192 | 1.1E-09 | 0.984 | 0.186 | 9.3E-08 |
| rs1517572 | 11:28829882 | METT5D1 | C/A | 0.581 | 0.99 | 47 | 0.879 | 0.138 | 1.5E-10 | 0.810 | 0.126 | 1.7E-10 |
| rs4592416 | 11:43800474 | HSD17B12 | G/A | 0.464 | 1.00 | 48 | 0.881 | 0.136 | 9.3E-11 | 0.798 | 0.126 | 2.3E-10 |
| rs11602180 | 11:48162453 | PTPRJ | C/T | 0.837 | 1.00 | 41 | 1.095 | 0.184 | 2.3E-09 | 0.918 | 0.168 | 5.8E-08 |
| rs174560 | 11:61581764 | FADS1 | C/T | 0.314 | 1.00 | 35 | 0.815 | 0.146 | 2.8E-08 | 0.792 | 0.138 | 5.8E-09 |
| rs12791153 | 11:80685181 | LOC729790 | T/A | 0.081 | 0.96 | 37 | 1.413 | 0.253 | 1.9E-08 | 1.320 | 0.234 | 2.6E-08 |
| rs1553132 | 11:88297740 | GRM5 | G/A | 0.258 | 1.00 | 36 | 0.870 | 0.155 | 2.5E-08 | 0.852 | 0.144 | 5.1E-09 |
| rs11614986 | 12:110007939 | MMAB | A/G | 0.821 | 1.00 | 35 | 0.983 | 0.177 | 2.7E-08 | 0.942 | 0.162 | 9.7E-09 |
| rs4767550 | 12:117951150 | KSR2 | G/A | 0.414 | 0.99 | 44 | 0.858 | 0.139 | 6.3E-10 | 0.822 | 0.126 | 1.1E-10 |
| rs34354917 | 12:38764559 | ALG10B | C/A | 0.710 | 0.99 | 35 | 0.825 | 0.150 | 3.9E-08 | 0.666 | 0.138 | 1.8E-06 |
| rs6575005 | 14:26954078 | NOVA1 | T/C | 0.758 | 1.00 | 40 | 0.934 | 0.159 | 4.4E-09 | 0.858 | 0.144 | 4.3E-09 |
| rs10483350 | 14:29816155 | MIR548AI | G/A | 0.195 | 0.99 | 42 | 1.042 | 0.172 | 1.5E-09 | 0.858 | 0.156 | 6.4E-08 |
| rs61985058 | 14:60233841 | RTN1 | T/C | 0.143 | 1.00 | 38 | 1.116 | 0.194 | 1.3E-08 | 0.936 | 0.180 | 2.1E-07 |
| rs55658675 | 14:65554638 | MAX | C/T | 0.645 | 1.00 | 35 | 0.788 | 0.142 | 2.0E-08 | 0.720 | 0.132 | 4.9E-08 |
| rs11621908 | 14:78495761 | ADCK1 | C/T | 0.917 | 0.97 | 39 | 1.446 | 0.250 | 5.6E-09 | 1.266 | 0.228 | 3.4E-08 |
| rs8038326 | 15:47989799 | SEMA6D | A/G | 0.727 | 1.00 | 45 | 0.955 | 0.152 | 2.8E-10 | 0.906 | 0.138 | 1.2E-10 |
| rs11643715 | 16:23909538 | PRKCB | G/C | 0.291 | 0.99 | 36 | 0.834 | 0.150 | 3.2E-08 | 0.660 | 0.144 | 5.6E-06 |
| rs9940646 | 16:53800629 | FTO | C/G | 0.578 | 1.00 | 63 | 1.017 | 0.137 | 1.2E-13 | 1.017 | 0.137 | 1.2E-13 |
| rs8050478 | 16:56120461 | GNAO1 | G/A | 0.500 | 1.00 | 57 | 0.960 | 0.136 | 1.7E-12 | 0.948 | 0.126 | 3.4E-14 |
| rs3095508 | 16:6550400 | RBFOX1 | C/A | 0.594 | 1.00 | 51 | 0.921 | 0.138 | 3.1E-11 | 0.702 | 0.126 | 3.4E-08 |
| rs205024 | 17:11227352 | SHISA6 | T/C | 0.384 | 1.00 | 40 | 0.830 | 0.140 | 3.9E-09 | 0.762 | 0.126 | 3.0E-09 |
| rs2139261 | 17:21313223 | KCNJ12 | G/C | 0.749 | 0.81 | 59 | 1.122 | 0.174 | 8.5E-11 | 1.122 | 0.174 | 8.5E-11 |
| rs1991556 | 17:44083402 | MAPT | G/A | 0.774 | 0.99 | 43 | 0.994 | 0.163 | 1.0E-09 | 0.994 | 0.163 | 1.0E-09 |
| rs9903973 | 17:50571227 | CA10 | C/T | 0.467 | 1.00 | 36 | 0.766 | 0.136 | 2.6E-08 | 0.750 | 0.132 | 1.2E-08 |
| rs7503199 | 17:8134275 | PER1 | C/T | 0.734 | 1.00 | 38 | 0.885 | 0.154 | 1.0E-08 | 0.810 | 0.138 | 6.9E-09 |
| rs12607679 | 18:53059748 | TCF4 | T/C | 0.738 | 0.98 | 70 | 1.208 | 0.156 | 8.3E-15 | 1.208 | 0.156 | 8.3E-15 |
| rs10421649 | 19:9942262 | FBXL12 | A/T | 0.557 | 0.98 | 39 | 0.798 | 0.138 | 6.9E-09 | 0.906 | 0.126 | 5.8E-13 |
| rs7556815 | 2:114085785 | PAX8 | A/G | 0.219 | 0.99 | 253 | 2.443 | 0.164 | 1.3E-49 | 2.352 | 0.150 | 1.8E-54 |
| rs12611523 | 2:139195328 | SPOPL | A/G | 0.545 | 0.99 | 35 | 0.758 | 0.137 | 3.1E-08 | 0.702 | 0.126 | 2.6E-08 |
| rs4128364 | 2:147612734 | PABPCP2 | C/T | 0.339 | 1.00 | 43 | 0.876 | 0.143 | 1.4E-09 | 0.780 | 0.132 | 3.9E-09 |
| rs4538155 | 2:157040773 | NR4A2 | T/C | 0.647 | 0.99 | 34 | 0.779 | 0.142 | 3.6E-08 | 0.762 | 0.132 | 5.8E-09 |
| rs11885663 | 2:166944004 | SCN1A | T/C | 0.248 | 1.00 | 44 | 0.973 | 0.157 | 8.6E-10 | 0.864 | 0.144 | 3.5E-09 |
| rs10173260 | 2:210377845 | MAP2 | C/T | 0.606 | 1.00 | 35 | 0.770 | 0.139 | 2.9E-08 | 0.618 | 0.126 | 1.6E-06 |
| rs374153 | 2:40382712 | SLC8A1 | C/T | 0.158 | 1.00 | 37 | 1.057 | 0.186 | 9.1E-09 | 0.990 | 0.174 | 8.5E-09 |
| rs75539574 | 2:58871658 | VRK2 | C/A | 0.086 | 0.98 | 92 | 2.175 | 0.244 | 6.9E-19 | 1.548 | 0.174 | 3.0E-19 |
| rs72804080 | 2:59358659 | LINC01122 | G/A | 0.150 | 0.98 | 36 | 1.068 | 0.192 | 2.9E-08 | 1.068 | 0.192 | 2.9E-08 |
| rs62120041 | 2:9185564 | MBOAT2 | T/C | 0.934 | 0.99 | 38 | 1.567 | 0.274 | 9.6E-09 | 1.380 | 0.270 | 3.2E-07 |
| rs2072727 | 20:43538733 | YWHAB | T/C | 0.436 | 1.00 | 39 | 0.795 | 0.137 | 7.9E-09 | 0.696 | 0.126 | 3.8E-08 |
| rs7644809 | 3:107564459 | BBX | T/C | 0.422 | 0.99 | 37 | 0.784 | 0.138 | 1.6E-08 | 0.624 | 0.126 | 1.0E-06 |
| rs13088093 | 3:135838598 | PPP2R3A | G/T | 0.336 | 0.99 | 53 | 0.976 | 0.144 | 7.0E-12 | 0.906 | 0.132 | 8.4E-12 |
| rs7616632 | 3:137031237 | IL20RB | T/G | 0.522 | 0.99 | 39 | 0.792 | 0.136 | 4.3E-09 | 0.732 | 0.126 | 4.8E-09 |
| rs112230981 | 3:55879269 | ERC2 | A/G | 0.950 | 0.98 | 42 | 1.892 | 0.314 | 2.2E-09 | 0.990 | 0.282 | 5.1E-04 |
| rs17732997 | 3:70470834 | FOXP1 | C/G | 0.569 | 0.99 | 37 | 0.776 | 0.137 | 1.2E-08 | 0.636 | 0.126 | 5.2E-07 |
| rs13109404 | 4:102896591 | BANK1 | T/G | 0.928 | 0.99 | 58 | 1.872 | 0.264 | 1.4E-12 | 1.716 | 0.264 | 9.5E-11 |
| rs2192528 | 4:18327896 | LCORL | A/G | 0.480 | 1.00 | 40 | 0.802 | 0.136 | 2.7E-09 | 0.666 | 0.126 | 9.9E-08 |
| rs17427571 | 4:82254908 | PRKG2 | A/G | 0.684 | 1.00 | 37 | 0.830 | 0.146 | 1.3E-08 | 0.726 | 0.132 | 7.4E-08 |
| rs35531607 | 4:92533225 | CCSER1 | C/T | 0.474 | 1.00 | 37 | 0.770 | 0.136 | 1.5E-08 | 0.708 | 0.126 | 2.2E-08 |
| rs56372231 | 5:102321905 | PAM | T/C | 0.334 | 1.00 | 57 | 1.017 | 0.144 | 2.2E-12 | 0.888 | 0.132 | 1.8E-11 |
| rs180769 | 5:135615615 | TRPC7 | T/C | 0.425 | 0.99 | 35 | 0.763 | 0.138 | 2.3E-08 | 0.678 | 0.126 | 1.1E-07 |
| rs11567976 | 5:137654218 | CDC25C | T/C | 0.571 | 1.00 | 36 | 0.768 | 0.137 | 2.1E-08 | 0.660 | 0.126 | 2.1E-07 |
| rs365663 | 5:1428883 | SLC6A3 | A/G | 0.546 | 0.99 | 47 | 0.878 | 0.137 | 1.0E-10 | 0.696 | 0.126 | 3.2E-08 |
| rs151014368 | 5:176751059 | LMAN2 | A/G | 0.206 | 0.98 | 38 | 0.966 | 0.169 | 9.1E-09 | 0.756 | 0.150 | 4.1E-07 |
| rs460692 | 5:3126584 | LINC01377 | C/T | 0.137 | 0.97 | 47 | 1.263 | 0.200 | 3.6E-10 | 1.206 | 0.204 | 3.2E-09 |
| rs34556183 | 6:28584775 | ZBED9 | A/G | 0.720 | 1.00 | 51 | 1.015 | 0.151 | 2.3E-11 | 0.990 | 0.150 | 2.5E-11 |
| rs80193650 | 6:33464363 | ZBTB9 | G/A | 0.162 | 1.00 | 34 | 1.010 | 0.184 | 4.1E-08 | 0.774 | 0.180 | 2.3E-05 |
| rs113113059 | 6:43160375 | CUL9 | T/C | 0.780 | 1.00 | 40 | 0.968 | 0.164 | 8.4E-09 | 0.828 | 0.156 | 8.5E-08 |
| rs9382445 | 6:54937974 | FAM83B | T/C | 0.623 | 1.00 | 44 | 0.872 | 0.140 | 4.8E-10 | 0.846 | 0.132 | 6.3E-11 |
| rs2231265 | 6:89790201 | PNRC1 | G/A | 0.772 | 1.00 | 35 | 0.897 | 0.162 | 2.7E-08 | 0.930 | 0.150 | 4.3E-10 |
| rs9345234 | 6:93162639 | LOC100129847 | C/A | 0.578 | 0.99 | 37 | 0.781 | 0.138 | 1.8E-08 | 0.714 | 0.126 | 2.1E-08 |
| rs2079070 | 7:114126432 | FOXP2 | C/G | 0.265 | 1.00 | 54 | 1.053 | 0.154 | 7.5E-12 | 1.053 | 0.154 | 7.5E-12 |
| rs7806045 | 7:132610266 | CHCHD3 | T/C | 0.755 | 1.00 | 36 | 0.887 | 0.158 | 1.4E-08 | 0.894 | 0.144 | 1.1E-09 |
| rs34731055 | 7:2106928 | MAD1L1 | T/C | 0.181 | 0.99 | 50 | 1.168 | 0.177 | 3.7E-11 | 1.044 | 0.162 | 1.1E-10 |
| rs73219758 | 8:14279446 | SGCZ | G/A | 0.708 | 1.00 | 50 | 0.984 | 0.150 | 5.6E-11 | 0.906 | 0.138 | 4.6E-11 |
| rs330088 | 8:9149746 | PPP1R3B | C/T | 0.547 | 0.99 | 46 | 0.868 | 0.137 | 2.7E-10 | -0.666 | 0.126 | 1.1E-07 |
| rs1776776 | 9:140497072 | ARRDC1 | T/C | 0.874 | 0.99 | 39 | 1.198 | 0.205 | 4.9E-09 | 1.104 | 0.198 | 2.7E-08 |
| rs10973207 | 9:37100525 | EBLN3 | T/G | 0.158 | 0.99 | 50 | 1.226 | 0.187 | 6.0E-11 | 1.050 | 0.174 | 1.3E-09 |

SNP=single nucleotide polymorphism; E/A=effect/alternative alleles; EAF=effect allele frequency; Info=imputation quality score; F=R^2^(n-2)/(1-R^2^), n stands for the sample size. R^2^ =2 × minor allele frequency × (1 − minor allele frequency) × (β / SD)^2^; Beta=per allele effect on SD units; SE=standard error; P value=p-value for the genetic association.

Table 2. Characteristics of SNPs for long sleep duration instrument from GWAS meta-analysis

| SNP | Chromosome position | Nearest Gene | Alleles  (E/A) | EAF | Info | F-statistic | Beta | SE | P value | |
| --- | --- | --- | --- | --- | --- | --- | --- | --- | --- | --- |
| rs6737318 | 2:114083120 | PAX8 | A/G | 0.778 | 0.99 | 891 | -0.076 | 0.010 | | 3.40E-13 |
| rs75458655 | 11:118115331 | MPZL2 | C/T | 0.977 | 1.00 | 686 | -0.185 | 0.027 | | 5.40E-12 |
| rs17688916 | 17:43778680 | KIAA1267 | T/A | 0.796 | 0.96 | 731 | 0.071 | 0.011 | | 1.10E-11 |
| rs17817288 | 16:53807764 | FTO | A/G | 0.518 | 1.00 | 339 | 0.039 | 0.007 | | 8.90E-09 |
| rs549961083 | 5:58184093 | RAB3C | C/T | 0.999 | 0.87 | 364 | -0.534 | 0.093 | | 9.60E-09 |
| rs3751046 | 11:122828342 | C11orf63 | A/G | 0.853 | 0.99 | 550 | -0.070 | 0.012 | | 2.00E-08 |
| rs7534398 | 1:7767464 | CAMTA1 | T/A | 0.799 | 0.99 | 317 | -0.047 | 0.008 | | 2.10E-08 |
| rs10899257 | 11:76415209 | GUCY2E | G/A | 0.856 | 1.00 | 511 | -0.068 | 0.012 | | 4.60E-08 |

SNP=single nucleotide polymorphism; E/A=effect/alternative alleles; EAF=effect allele frequency; Info=imputation quality score; F= R^2^(n-2)/(1-R^2^), n stands for the sample size. R^2^=2 × minor allele frequency × (1 − minor allele frequency) × (β / SD)^2^; Beta=per allele effect on SD units; SE=standard error; P value=p-value for the genetic association.

Table 3. Characteristics of SNPs for short sleep duration instrument from GWAS meta-analysis

| SNP | Chromosome position | Nearest Gene | Alleles  (E/A) | EAF | Info | F-statistic | Beta | SE | P value |
| --- | --- | --- | --- | --- | --- | --- | --- | --- | --- |
| rs2820313 | 1:201870221 | LMOD1 | A/G | 0.659 | 1.00 | 187 | -0.031 | 0.006 | 2.30E-09 |
| rs7524118 | 1:34736052 | CSMD2 | T/C | 0.292 | 1.00 | 161 | -0.030 | 0.006 | 4.90E-08 |
| rs2186122 | 1:66470206 | PDE4B | A/T | 0.438 | 0.99 | 124 | -0.024 | 0.006 | 4.80E-09 |
| rs12567114 | 1:98527951 | DPYD | G/A | 0.725 | 0.99 | 235 | 0.036 | 0.007 | 4.10E-09 |
| rs1607227 | 11:28808617 | METT5D1 | G/T | 0.705 | 0.99 | 173 | 0.031 | 0.007 | 1.50E-09 |
| rs7939345 | 11:47980568 | PTPRJ | T/G | 0.208 | 1.00 | 184 | 0.035 | 0.007 | 4.00E-08 |
| rs17388803 | 15:48027204 | SEMA6D | A/C | 0.894 | 0.96 | 233 | -0.053 | 0.010 | 6.50E-10 |
| rs59779556 | 16:56227965 | GNAO1 | T/G | 0.554 | 1.00 | 134 | 0.025 | 0.006 | 2.00E-08 |
| rs205024 | 17:11227352 | SHISA6 | C/T | 0.617 | 1.00 | 197 | 0.031 | 0.006 | 2.70E-08 |
| rs12963463 | 18:53099093 | TCF4 | C/T | 0.299 | 0.97 | 153 | 0.029 | 0.006 | 1.90E-11 |
| rs2863957 | 2:114089551 | PAX8 | C/A | 0.782 | 1.00 | 453 | 0.054 | 0.007 | 2.60E-18 |
| rs1380703 | 2:57941287 | VRK2 | A/G | 0.616 | 0.96 | 264 | -0.035 | 0.006 | 1.60E-11 |
| rs75539574 | 2:58871658 | LINC01122 | A/C | 0.915 | 0.98 | 141 | 0.045 | 0.011 | 8.40E-11 |
| rs5757675 | 22:39838892 | MGAT3 | G/T | 0.260 | 0.99 | 203 | 0.034 | 0.006 | 2.70E-09 |
| rs2014830 | 3:50172397 | RBM5 | C/T | 0.698 | 0.99 | 164 | 0.030 | 0.006 | 2.70E-08 |
| rs13107325 | 4:103188709 | SLC39A8 | C/T | 0.925 | 1.00 | 347 | -0.075 | 0.011 | 2.50E-13 |
| rs17005118 | 4:82288564 | RASGEF1B | G/A | 0.735 | 1.00 | 152 | -0.030 | 0.007 | 2.50E-09 |
| rs3776864 | 5:102327868 | PAM | A/C | 0.667 | 1.00 | 197 | 0.031 | 0.006 | 1.70E-08 |
| rs4585442 | 5:135508381 | SMAD5 | G/A | 0.311 | 1.00 | 178 | -0.031 | 0.006 | 8.10E-10 |
| rs12518468 | 5:7249696 | ADCY2 | T/C | 0.328 | 0.99 | 195 | -0.031 | 0.006 | 8.50E-09 |
| rs9321171 | 6:129848635 | LAMA2 | C/T | 0.540 | 0.99 | 220 | 0.031 | 0.006 | 4.20E-08 |
| rs142180737 | 6:28344731 | ZSCAN12 | T/C | 0.991 | 0.86 | 200 | -0.154 | 0.032 | 4.40E-09 |
| rs12661667 | 6:41792545 | USP49 | C/T | 0.737 | 1.00 | 132 | -0.028 | 0.007 | 2.80E-08 |
| rs9367621 | 6:55040290 | HCRTR2 | T/A | 0.431 | 1.00 | 123 | 0.024 | 0.006 | 1.60E-08 |
| rs1229762 | 7:114218582 | FOXP2 | C/T | 0.665 | 1.00 | 277 | -0.037 | 0.006 | 1.00E-12 |
| rs11763750 | 7:2080114 | MAD1L1 | G/A | 0.814 | 1.00 | 169 | 0.035 | 0.007 | 5.10E-09 |
| rs60882754 | 8:52886619 | PCMTD1 | A/T | 0.939 | 1.00 | 157 | 0.055 | 0.012 | 1.80E-08 |

SNP=single nucleotide polymorphism; E/A=effect/alternative alleles; EAF=effect allele frequency; Info=imputation quality score; R^2^=2×minor allele frequency×(1 − minor allele frequency)×(β/SD)^2^; Beta=per allele effect on SD units; SE=standard error; Beta=per allele effect on SD units; SE= standard error; P value=p-value for the genetic association.

Table 4. The raw information of continuous sleep duration instrument SNPs with AF and HF

| SNP | Alleles  (E/A) | Atrial Fibrillation | | | |  | Heart Failure | | | |
| --- | --- | --- | --- | --- | --- | --- | --- | --- | --- | --- |
|  |  | EAF | Beta | SE | P value |  | EAF | Beta | SE | P value |
| rs915416 | C/G | 0.698 | -0.0030 | 0.0073 | 0.677 |  | 0.293 | 0.0033 | 0.0086 | 0.704 |
| rs269054 | A/T | 0.565 | -0.0001 | 0.0067 | 0.983 |  | 0.435 | -0.0075 | 0.0079 | 0.345 |
| rs61796569 | T/C | 0.741 | -0.0012 | 0.0076 | 0.874 |  | 0.261 | -0.0009 | 0.0089 | 0.922 |
| rs12567114 | A/G | 0.722 | 0.0134 | 0.0075 | 0.076 |  | 0.277 | 0.0069 | 0.0088 | 0.436 |
| rs11190970 | G/A | 0.794 | 0.0277 | 0.0083 | 0.001 |  | 0.207 | -0.0097 | 0.0097 | 0.316 |
| rs7915425 | T/C | 0.808 | -0.0049 | 0.0088 | 0.575 |  | 0.175 | 0.0144 | 0.0103 | 0.163 |
| rs12246842 | A/G | 0.541 | -0.0102 | 0.0068 | 0.132 |  | 0.455 | -0.0031 | 0.0079 | 0.693 |
| rs10761674 | C/T | 0.483 | -0.0214 | 0.0066 | 0.001 |  | 0.522 | -0.0066 | 0.0078 | 0.396 |
| rs1939455 | G/T | 0.884 | 0.0002 | 0.0107 | 0.987 |  | 0.114 | 0.0077 | 0.0128 | 0.545 |
| rs7115226 | A/C | 0.923 | 0.0233 | 0.0127 | 0.067 |  | 0.076 | 0.0103 | 0.0150 | 0.493 |
| rs1263056 | A/G | 0.482 | 0.0047 | 0.0067 | 0.480 |  | 0.506 | -0.0216 | 0.0079 | 0.007 |
| rs7951019 | G/T | 0.031 | 0.0269 | 0.0213 | 0.206 |  | 0.969 | -0.0112 | 0.0251 | 0.656 |
| rs1057703 | G/T | 0.146 | 0.0118 | 0.0093 | 0.207 |  | 0.853 | 0.0132 | 0.0110 | 0.233 |
| rs1517572 | C/A | 0.588 | -0.0127 | 0.0068 | 0.063 |  | 0.405 | -0.0117 | 0.0080 | 0.142 |
| rs4592416 | G/A | 0.463 | -0.0089 | 0.0066 | 0.182 |  | 0.536 | -0.0220 | 0.0078 | 0.005 |
| rs11602180 | C/T | 0.841 | -0.0016 | 0.0090 | 0.861 |  | 0.159 | -0.0184 | 0.0107 | 0.086 |
| rs174560 | C/T | 0.320 | -0.0136 | 0.0071 | 0.056 |  | 0.678 | -0.0201 | 0.0084 | 0.017 |
| rs12791153 | T/A | 0.090 | -0.0087 | 0.0125 | 0.486 |  | 0.912 | -0.0171 | 0.0145 | 0.236 |
| rs1553132 | G/A | 0.263 | -0.0087 | 0.0075 | 0.248 |  | 0.739 | -0.0094 | 0.0090 | 0.296 |
| rs11614986 | A/G | 0.177 | 0.0193 | 0.0087 | 0.026 |  | 0.825 | 0.0104 | 0.0103 | 0.311 |
| rs4767550 | G/A | 0.417 | -0.0101 | 0.0068 | 0.138 |  | 0.583 | -0.0021 | 0.0079 | 0.789 |
| rs34354917 | C/A | 0.710 | 0.0007 | 0.0079 | 0.925 |  | 0.288 | 0.0031 | 0.0092 | 0.740 |
| rs6575005 | T/C | 0.235 | 0.0056 | 0.0078 | 0.473 |  | 0.768 | -0.0002 | 0.0093 | 0.985 |
| rs10483350 | G/A | 0.193 | 0.0174 | 0.0085 | 0.041 |  | 0.807 | -0.0110 | 0.0102 | 0.280 |
| rs61985058 | T/C | 0.862 | -0.0108 | 0.0100 | 0.280 |  | 0.134 | -0.0137 | 0.0117 | 0.242 |
| rs55658675 | C/T | 0.647 | 0.0192 | 0.0070 | 0.006 |  | 0.356 | -0.0072 | 0.0082 | 0.380 |
| rs11621908 | C/T | 0.921 | 0.0046 | 0.0124 | 0.709 |  | 0.080 | -0.0208 | 0.0146 | 0.153 |
| rs8038326 | A/G | 0.278 | 0.0114 | 0.0075 | 0.126 |  | 0.714 | 0.0119 | 0.0087 | 0.170 |
| rs11643715 | G/C | 0.283 | -0.0015 | 0.0074 | 0.835 |  | 0.721 | -0.0062 | 0.0087 | 0.481 |
| rs9940646 | C/G | 0.433 | -0.0281 | 0.0067 | 2.61E-05 |  | 0.565 | -0.0439 | 0.0079 | 2.64E-08 |
| rs8050478 | G/A | 0.514 | 0.0060 | 0.0067 | 0.369 |  | 0.485 | 0.0083 | 0.0078 | 0.290 |
| rs3095508 | C/A | 0.606 | 0.0024 | 0.0068 | 0.718 |  | 0.393 | 0.0021 | 0.0081 | 0.793 |
| rs205024 | T/C | 0.614 | -0.0059 | 0.0068 | 0.383 |  | 0.390 | -0.0113 | 0.0080 | 0.159 |
| rs2139261 | G/C | 0.740 | 0.0030 | 0.0097 | 0.754 |  | NA | NA | NA | NA |
| rs1991556 | G/A | 0.786 | -0.0489 | 0.0086 | 1.25E-08 |  | 0.205 | -0.0125 | 0.0102 | 0.220 |
| rs9903973 | C/T | 0.469 | -0.0059 | 0.0066 | 0.374 |  | 0.532 | -0.0029 | 0.0079 | 0.712 |
| rs7503199 | C/T | 0.741 | 0.0089 | 0.0075 | 0.240 |  | 0.258 | 0.0064 | 0.0089 | 0.472 |
| rs12607679 | T/C | 0.250 | 0.0065 | 0.0077 | 0.395 |  | 0.754 | -0.0163 | 0.0091 | 0.073 |
| rs10421649 | A/T | 0.452 | -0.0035 | 0.0069 | 0.609 |  | 0.551 | -0.0110 | 0.0080 | 0.167 |
| rs7556815 | A/G | 0.776 | -0.0101 | 0.0080 | 0.209 |  | 0.226 | -0.0090 | 0.0094 | 0.336 |
| rs12611523 | A/G | 0.443 | -0.0061 | 0.0067 | 0.367 |  | 0.559 | -0.0158 | 0.0079 | 0.046 |
| rs4128364 | C/T | 0.346 | -0.0171 | 0.0070 | 0.014 |  | 0.649 | -0.0103 | 0.0083 | 0.214 |
| rs4538155 | T/C | 0.357 | -0.0002 | 0.0069 | 0.981 |  | 0.651 | -0.0007 | 0.0082 | 0.931 |
| rs11885663 | T/C | 0.751 | 0.0000 | 0.0078 | 0.996 |  | 0.251 | -0.0127 | 0.0090 | 0.161 |
| rs10173260 | C/T | 0.602 | -0.0017 | 0.0068 | 0.805 |  | 0.396 | -0.0002 | 0.0080 | 0.980 |
| rs374153 | C/T | 0.174 | 0.0058 | 0.0092 | 0.530 |  | 0.845 | -0.0070 | 0.0111 | 0.526 |
| rs75539574 | C/A | 0.088 | 0.0068 | 0.0120 | 0.571 |  | 0.912 | 0.0209 | 0.0141 | 0.139 |
| rs72804080 | G/A | 0.158 | -0.0140 | 0.0094 | 0.137 |  | 0.839 | -0.0064 | 0.0109 | 0.559 |
| rs62120041 | T/C | 0.061 | -0.0284 | 0.0145 | 0.049 |  | 0.937 | 0.0177 | 0.0166 | 0.284 |
| rs2072727 | T/C | 0.558 | 0.0021 | 0.0067 | 0.759 |  | 0.436 | -0.0137 | 0.0079 | 0.083 |
| rs7644809 | T/C | 0.572 | 0.0073 | 0.0068 | 0.280 |  | 0.419 | 0.0019 | 0.0080 | 0.810 |
| rs13088093 | G/T | 0.342 | -0.0204 | 0.0071 | 0.004 |  | 0.662 | -0.0003 | 0.0083 | 0.971 |
| rs7616632 | T/G | 0.473 | -0.0117 | 0.0067 | 0.082 |  | 0.532 | 0.0010 | 0.0079 | 0.896 |
| rs112230981 | A/G | 0.050 | -0.0037 | 0.0166 | 0.824 |  | 0.952 | -0.0087 | 0.0192 | 0.651 |
| rs17732997 | C/G | 0.423 | 0.0014 | 0.0068 | 0.840 |  | 0.580 | -0.0077 | 0.0080 | 0.333 |
| rs13109404 | T/G | 0.065 | -0.0031 | 0.0152 | 0.837 |  | 0.937 | -0.0507 | 0.0178 | 0.004 |
| rs2192528 | A/G | 0.514 | -0.0076 | 0.0067 | 0.254 |  | 0.485 | -0.0150 | 0.0078 | 0.056 |
| rs17427571 | A/G | 0.325 | -0.0312 | 0.0071 | 1.05E-05 |  | 0.667 | -0.0130 | 0.0083 | 0.114 |
| rs35531607 | C/T | 0.456 | 0.0044 | 0.0067 | 0.511 |  | 0.542 | 0.0070 | 0.0078 | 0.370 |
| rs56372231 | T/C | 0.671 | 0.0175 | 0.0070 | 0.013 |  | 0.326 | -0.0048 | 0.0083 | 0.567 |
| rs180769 | T/C | 0.574 | 0.0084 | 0.0068 | 0.215 |  | 0.562 | -0.0026 | 0.0079 | 0.742 |
| rs11567976 | T/C | 0.432 | 0.0138 | 0.0067 | 0.040 |  | 0.562 | -0.0026 | 0.0079 | 0.742 |
| rs365663 | A/G | 0.446 | 0.0019 | 0.0067 | 0.780 |  | 0.540 | 0.0010 | 0.0080 | 0.896 |
| rs151014368 | A/G | 0.774 | 0.0205 | 0.0082 | 0.012 |  | 0.230 | 0.0026 | 0.0095 | 0.785 |
| rs460692 | C/T | 0.172 | 0.0000 | 0.0096 | 0.998 |  | 0.843 | 0.0095 | 0.0111 | 0.391 |
| rs34556183 | A/G | 0.258 | 0.0024 | 0.0081 | 0.762 |  | 0.753 | -0.0006 | 0.0099 | 0.951 |
| rs80193650 | G/A | 0.157 | 0.0012 | 0.0093 | 0.900 |  | 0.844 | -0.0153 | 0.0109 | 0.161 |
| rs113113059 | T/C | 0.213 | 0.0012 | 0.0081 | 0.883 |  | 0.790 | -0.0054 | 0.0097 | 0.574 |
| rs9382445 | T/C | 0.359 | -0.0025 | 0.0069 | 0.721 |  | 0.642 | -0.0020 | 0.0082 | 0.804 |
| rs2231265 | G/A | 0.754 | 0.0051 | 0.0079 | 0.520 |  | 0.225 | -0.0051 | 0.0093 | 0.583 |
| rs9345234 | C/A | 0.564 | 0.0021 | 0.0067 | 0.749 |  | 0.434 | -0.0009 | 0.0078 | 0.908 |
| rs2079070 | C/G | 0.724 | -0.0062 | 0.0077 | 0.421 |  | 0.261 | -0.0053 | 0.0090 | 0.558 |
| rs7806045 | T/C | 0.255 | 0.0010 | 0.0077 | 0.893 |  | 0.743 | -0.0075 | 0.0090 | 0.402 |
| rs34731055 | T/C | 0.819 | -0.0183 | 0.0086 | 0.034 |  | 0.196 | -0.0232 | 0.0101 | 0.022 |
| rs73219758 | G/A | 0.714 | -0.0041 | 0.0074 | 0.580 |  | 0.284 | 0.0038 | 0.0087 | 0.661 |
| rs330088 | C/T | 0.539 | -0.0100 | 0.0068 | 0.141 |  | 0.467 | 0.0079 | 0.0082 | 0.334 |
| rs1776776 | T/C | 0.133 | -0.0125 | 0.0097 | 0.198 |  | 0.855 | -0.0111 | 0.0114 | 0.328 |
| rs10973207 | T/G | 0.838 | -0.0034 | 0.0092 | 0.715 |  | 0.170 | -0.0031 | 0.0106 | 0.768 |

SNP=single nucleotide polymorphism; E/A=effect/alternative alleles; EAF=effect allele frequency; Beta = per allele effect on SD units; SE = standard error; P value = p-value for the genetic association.

Table 5. The raw information of long sleep duration instrument SNPs with AF and HF

| SNP | Alleles  (E/A) | Atrial Fibrillation | | | | |  | Heart Failure | | | |
| --- | --- | --- | --- | --- | --- | --- | --- | --- | --- | --- | --- |
|  |  | EAF | Beta | SE | P value | |  | EAF | Beta | SE | P value |
| rs6737318 | A/G | 0.224 | 0.0115 | 0.0080 | | 0.152 |  | 0.776 | 0.0093 | 0.0094 | 0.323 |
| rs75458655 | C/T | 0.978 | -0.0349 | 0.0252 | | 0.166 |  | 0.022 | 0.0121 | 0.0296 | 0.684 |
| rs17688916 | T/A | 0.807 | -0.0480 | 0.0091 | | 1.41E-07 |  | 0.187 | -0.0133 | 0.011 | 0.224 |
| rs17817288 | A/G | 0.487 | -0.0208 | 0.0066 | | 0.002 |  | 0.508 | -0.0348 | 0.0078 | 8.88E-06 |
| rs549961083 | C/T | 0.998 | 0.0435 | 0.1115 | | 0.696 |  | NA | NA | NA | NA |
| rs3751046 | A/G | 0.146 | -0.0118 | 0.0094 | | 0.208 |  | 0.853 | -0.012 | 0.0112 | 0.286 |
| rs7534398 | T/A | 0.802 | -0.0057 | 0.0085 | | 0.497 |  | 0.194 | 0.0148 | 0.01 | 0.141 |
| rs10899257 | G/A | 0.856 | 0.0054 | 0.0095 | | 0.566 |  | 0.145 | 0.0023 | 0.0112 | 0.837 |

SNP=single nucleotide polymorphism; E/A=effect/alternative alleles; EAF=effect allele frequency; Beta = per allele effect on SD units; SE = standard error; P value = p-value for the genetic association.

Table 6. The raw information of short sleep duration instrument SNPs with AF and HF

| SNP | Alleles  (E/A) | Atrial Fibrillation | | | |  | Heart Failure | | | |
| --- | --- | --- | --- | --- | --- | --- | --- | --- | --- | --- |
|  |  | EAF | Beta | SE | P value |  | EAF | Beta | SE | P value |
| rs2820313 | A/G | 0.341 | -0.0095 | 0.0070 | 0.174 |  | 0.664 | -0.0122 | 0.0083 | 0.142 |
| rs7524118 | T/C | 0.694 | -0.0006 | 0.0073 | 0.937 |  | 0.296 | 0.0014 | 0.0085 | 0.866 |
| rs2186122 | A/T | 0.561 | -0.0113 | 0.0067 | 0.091 |  | 0.432 | -0.0014 | 0.0079 | 0.861 |
| rs12567114 | G/A | 0.722 | -0.0134 | 0.0075 | 0.076 |  | 0.277 | -0.0069 | 0.0088 | 0.436 |
| rs1607227 | G/T | 0.696 | 0.0114 | 0.0073 | 0.121 |  | 0.298 | 0.0037 | 0.0086 | 0.671 |
| rs7939345 | T/G | 0.785 | -0.0006 | 0.0085 | 0.942 |  | 0.196 | -0.0003 | 0.0100 | 0.980 |
| rs17388803 | A/C | 0.114 | 0.0167 | 0.0109 | 0.126 |  | 0.882 | 0.0071 | 0.0125 | 0.571 |
| rs59779556 | T/G | 0.460 | -0.0025 | 0.0067 | 0.703 |  | 0.537 | -0.0015 | 0.0079 | 0.848 |
| rs205024 | C/T | 0.614 | 0.0059 | 0.0068 | 0.383 |  | 0.390 | 0.0113 | 0.0080 | 0.159 |
| rs12963463 | C/T | 0.304 | -0.0066 | 0.0074 | 0.368 |  | 0.710 | 0.0113 | 0.0087 | 0.194 |
| rs2863957 | C/A | 0.776 | 0.0105 | 0.0080 | 0.189 |  | 0.225 | 0.0112 | 0.0095 | 0.241 |
| rs1380703 | A/G | 0.384 | -0.0061 | 0.0071 | 0.390 |  | 0.617 | 0.0016 | 0.0083 | 0.846 |
| rs75539574 | A/C | 0.088 | -0.0068 | 0.0120 | 0.571 |  | 0.912 | -0.0209 | 0.0141 | 0.139 |
| rs5757675 | G/T | 0.272 | 0.0144 | 0.0077 | 0.064 |  | 0.736 | 0.0013 | 0.0089 | 0.883 |
| rs2014830 | C/T | 0.696 | 0.0124 | 0.0072 | 0.088 |  | 0.312 | 0.0087 | 0.0085 | 0.306 |
| rs13107325 | C/T | 0.930 | -0.0130 | 0.0142 | 0.360 |  | 0.071 | -0.0701 | 0.0170 | 3.97E-05 |
| rs17005118 | G/A | 0.729 | -0.0294 | 0.0075 | 8.80E-05 |  | 0.270 | -0.0174 | 0.0088 | 0.048 |
| rs3776864 | A/C | 0.328 | -0.0172 | 0.0072 | 0.017 |  | 0.683 | 0.0021 | 0.0085 | 0.802 |
| rs4585442 | G/A | 0.309 | 0.0109 | 0.0073 | 0.132 |  | 0.697 | -0.0019 | 0.0085 | 0.828 |
| rs12518468 | T/C | 0.331 | 0.0028 | 0.0071 | 0.692 |  | 0.675 | -0.0006 | 0.0084 | 0.945 |
| rs9321171 | C/T | 0.542 | -0.0039 | 0.0067 | 0.566 |  | 0.452 | -0.0058 | 0.0079 | 0.461 |
| rs142180737 | T/C | 0.011 | -0.0348 | 0.0433 | 0.421 |  | NA | NA | NA | NA |
| rs12661667 | C/T | 0.742 | -0.0028 | 0.0076 | 0.714 |  | 0.254 | 0.0126 | 0.0090 | 0.162 |
| rs9367621 | T/A | 0.419 | 0.0061 | 0.0067 | 0.365 |  | 0.584 | 0.0058 | 0.0079 | 0.462 |
| rs1229762 | C/T | 0.339 | -0.0073 | 0.0071 | 0.304 |  | 0.667 | -0.0047 | 0.0083 | 0.574 |
| rs11763750 | G/A | 0.815 | 0.0226 | 0.0085 | 0.008 |  | 0.199 | 0.0226 | 0.0100 | 0.023 |
| rs60882754 | A/T | 0.056 | 0.0088 | 0.0147 | 0.550 |  | 0.946 | -0.0076 | 0.0178 | 0.668 |

SNP=single nucleotide polymorphism; E/A=effect/alternative alleles; EAF=effect allele frequency; Beta = per allele effect on SD units; SE = standard error; P value = p-value for the genetic association.

Table 7. The information of continuous sleep duration instrument SNPs with AF and HF in single sex

| SNP | Alleles  (E/A) | AF in male | | | AF in female | | | HF in male | | | HF in female | | |
| --- | --- | --- | --- | --- | --- | --- | --- | --- | --- | --- | --- | --- | --- |
|  |  | Beta | SE | P value | Beta | SE | P value | Beta | SE | P value | Beta | SE | P value |
| rs915416 | C/G | -2.19E-04 | 6.23E-04 | 0.724 | -1.73E-04 | 3.68E-04 | 0.639 | -4.00E-04 | 2.91E-04 | 0.169 | 1.90E-04 | 1.57E-04 | 0.226 |
| rs269054 | A/T | 5.01E-04 | 5.73E-04 | 0.382 | -4.99E-04 | 3.38E-04 | 0.140 | -1.94E-04 | 2.68E-04 | 0.470 | -1.42E-05 | 1.44E-04 | 0.921 |
| rs61796569 | T/C | -3.39E-04 | 6.39E-04 | 0.596 | -1.06E-04 | 3.79E-04 | 0.780 | 2.13E-04 | 2.99E-04 | 0.476 | -3.83E-05 | 1.62E-04 | 0.813 |
| rs12567114 | A/G | 8.68E-04 | 6.35E-04 | 0.171 | 2.22E-04 | 3.75E-04 | 0.554 | 9.09E-05 | 2.97E-04 | 0.759 | -1.61E-04 | 1.60E-04 | 0.315 |
| rs11190970 | G/A | 9.91E-04 | 7.05E-04 | 0.160 | 4.62E-05 | 4.17E-04 | 0.912 | 2.66E-04 | 3.30E-04 | 0.420 | 1.82E-04 | 1.78E-04 | 0.305 |
| rs7915425 | T/C | -7.62E-05 | 7.48E-04 | 0.919 | 2.10E-04 | 4.42E-04 | 0.635 | 2.73E-04 | 3.49E-04 | 0.434 | 2.12E-05 | 1.88E-04 | 0.910 |
| rs12246842 | A/G | -6.79E-04 | 5.68E-04 | 0.232 | -7.71E-05 | 3.35E-04 | 0.818 | -1.62E-04 | 2.65E-04 | 0.542 | -9.00E-06 | 1.43E-04 | 0.950 |
| rs10761674 | C/T | -1.03E-03 | 5.65E-04 | 0.069 | -1.66E-05 | 3.34E-04 | 0.960 | -1.18E-04 | 2.64E-04 | 0.656 | 1.26E-04 | 1.43E-04 | 0.375 |
| rs1939455 | G/T | 4.57E-04 | 8.88E-04 | 0.607 | 1.89E-04 | 5.27E-04 | 0.720 | -4.80E-04 | 4.15E-04 | 0.248 | 1.41E-04 | 2.25E-04 | 0.530 |
| rs7115226 | A/C | 1.82E-03 | 1.09E-03 | 0.095 | 1.56E-04 | 6.43E-04 | 0.808 | -4.90E-04 | 5.09E-04 | 0.335 | 3.89E-04 | 2.74E-04 | 0.156 |
| rs1263056 | A/G | 1.13E-03 | 5.68E-04 | 0.046 | -6.77E-05 | 3.36E-04 | 0.840 | -3.27E-04 | 2.66E-04 | 0.219 | -4.13E-04 | 1.43E-04 | 0.004 |
| rs7951019 | G/T | -1.17E-03 | 1.63E-03 | 0.473 | -6.51E-05 | 9.59E-04 | 0.946 | -8.13E-04 | 7.61E-04 | 0.285 | 3.74E-04 | 4.09E-04 | 0.361 |
| rs1057703 | G/T | 4.44E-04 | 8.00E-04 | 0.579 | 4.07E-04 | 4.73E-04 | 0.389 | -1.87E-04 | 3.74E-04 | 0.618 | -1.80E-04 | 2.02E-04 | 0.371 |
| rs1517572 | C/A | -3.05E-04 | 5.73E-04 | 0.594 | 1.00E-04 | 3.38E-04 | 0.767 | -4.66E-04 | 2.68E-04 | 0.082 | 1.47E-04 | 1.44E-04 | 0.309 |
| rs4592416 | G/A | -5.90E-04 | 5.67E-04 | 0.298 | -2.62E-04 | 3.34E-04 | 0.433 | -3.17E-04 | 2.65E-04 | 0.232 | -2.49E-04 | 1.43E-04 | 0.081 |
| rs11602180 | C/T | -2.35E-04 | 7.66E-04 | 0.759 | -2.45E-04 | 4.53E-04 | 0.588 | -9.25E-05 | 3.58E-04 | 0.796 | 9.35E-05 | 1.93E-04 | 0.628 |
| rs174560 | C/T | -2.28E-04 | 6.08E-04 | 0.708 | -3.18E-04 | 3.59E-04 | 0.377 | 4.21E-04 | 2.84E-04 | 0.138 | -2.08E-04 | 1.53E-04 | 0.174 |
| rs12791153 | T/A | 8.25E-04 | 1.05E-03 | 0.434 | 6.94E-04 | 6.21E-04 | 0.264 | 9.28E-04 | 4.93E-04 | 0.060 | -2.78E-04 | 2.65E-04 | 0.294 |
| rs1553132 | G/A | -3.94E-04 | 6.45E-04 | 0.541 | -7.94E-04 | 3.80E-04 | 0.037 | -1.88E-04 | 3.01E-04 | 0.534 | 2.12E-05 | 1.62E-04 | 0.896 |
| rs11614986 | A/G | 1.43E-04 | 7.37E-04 | 0.846 | 3.97E-04 | 4.34E-04 | 0.360 | -5.02E-04 | 3.45E-04 | 0.145 | 5.88E-05 | 1.85E-04 | 0.750 |
| rs4767550 | G/A | -1.44E-03 | 5.76E-04 | 0.012 | -2.13E-04 | 3.41E-04 | 0.532 | -4.98E-04 | 2.69E-04 | 0.065 | 2.51E-05 | 1.45E-04 | 0.863 |
| rs34354917 | C/A | -7.21E-04 | 6.24E-04 | 0.248 | 3.00E-05 | 3.69E-04 | 0.935 | 2.84E-04 | 2.92E-04 | 0.329 | -9.32E-05 | 1.57E-04 | 0.553 |
| rs6575005 | T/C | -2.14E-04 | 6.59E-04 | 0.746 | 1.95E-04 | 3.89E-04 | 0.616 | 2.09E-04 | 3.08E-04 | 0.498 | 1.39E-04 | 1.66E-04 | 0.402 |
| rs10483350 | G/A | 2.08E-04 | 7.14E-04 | 0.771 | -1.42E-05 | 4.24E-04 | 0.973 | 1.51E-04 | 3.34E-04 | 0.652 | 8.53E-05 | 1.81E-04 | 0.637 |
| rs61985058 | T/C | -3.35E-04 | 8.07E-04 | 0.678 | 4.84E-04 | 4.74E-04 | 0.307 | -5.05E-04 | 3.77E-04 | 0.181 | 1.79E-04 | 2.02E-04 | 0.377 |
| rs55658675 | C/T | -2.00E-04 | 5.90E-04 | 0.735 | 4.66E-04 | 3.49E-04 | 0.181 | 3.14E-04 | 2.76E-04 | 0.255 | 1.01E-04 | 1.49E-04 | 0.495 |
| rs11621908 | C/T | 5.78E-04 | 1.04E-03 | 0.580 | -1.37E-03 | 6.11E-04 | 0.025 | -6.95E-05 | 4.88E-04 | 0.887 | -5.44E-04 | 2.61E-04 | 0.037 |
| rs8038326 | A/G | 4.47E-04 | 6.35E-04 | 0.481 | 3.28E-04 | 3.74E-04 | 0.381 | 4.56E-04 | 2.97E-04 | 0.125 | 6.61E-05 | 1.60E-04 | 0.679 |
| rs11643715 | G/C | -1.56E-03 | 6.22E-04 | 0.012 | -2.22E-04 | 3.69E-04 | 0.546 | 8.69E-05 | 2.91E-04 | 0.765 | -1.76E-05 | 1.57E-04 | 0.911 |
| rs9940646 | C/G | 5.10E-04 | 5.72E-04 | 0.373 | -3.40E-04 | 3.38E-04 | 0.314 | 4.22E-05 | 2.67E-04 | 0.875 | -1.04E-04 | 1.44E-04 | 0.472 |
| rs8050478 | G/A | 6.04E-04 | 5.66E-04 | 0.286 | 2.32E-04 | 3.34E-04 | 0.487 | 3.57E-04 | 2.64E-04 | 0.176 | -4.84E-05 | 1.42E-04 | 0.734 |
| rs3095508 | C/A | -8.45E-04 | 5.74E-04 | 0.141 | 3.96E-05 | 3.40E-04 | 0.907 | 2.15E-04 | 2.68E-04 | 0.423 | -1.46E-04 | 1.45E-04 | 0.312 |
| rs205024 | T/C | -3.86E-04 | 5.81E-04 | 0.507 | 1.58E-05 | 3.43E-04 | 0.963 | -4.30E-06 | 2.71E-04 | 0.987 | -1.78E-04 | 1.46E-04 | 0.223 |
| rs2139261 | G/C | NA | NA | NA | NA | NA | NA | NA | NA | NA | NA | NA | NA |
| rs1991556 | G/A | -2.12E-03 | 6.79E-04 | 0.002 | -5.33E-04 | 4.01E-04 | 0.184 | 9.06E-05 | 3.17E-04 | 0.775 | 1.00E-04 | 1.71E-04 | 0.558 |
| rs9903973 | C/T | 4.01E-04 | 5.67E-04 | 0.479 | 1.96E-04 | 3.35E-04 | 0.559 | -3.79E-04 | 2.65E-04 | 0.152 | 2.48E-04 | 1.43E-04 | 0.083 |
| rs7503199 | C/T | 1.18E-04 | 6.40E-04 | 0.853 | 4.26E-04 | 3.77E-04 | 0.259 | -9.71E-05 | 2.99E-04 | 0.746 | -8.91E-05 | 1.61E-04 | 0.580 |
| rs12607679 | T/C | -2.96E-04 | 6.47E-04 | 0.647 | 9.06E-05 | 3.82E-04 | 0.812 | -4.06E-05 | 3.02E-04 | 0.893 | -2.10E-04 | 1.63E-04 | 0.197 |
| rs10421649 | A/T | -4.45E-04 | 5.73E-04 | 0.437 | 1.34E-04 | 3.38E-04 | 0.691 | -6.24E-04 | 2.68E-04 | 0.020 | 1.49E-04 | 1.44E-04 | 0.300 |
| rs7556815 | A/G | -9.60E-04 | 6.85E-04 | 0.161 | 4.44E-04 | 4.04E-04 | 0.272 | -2.77E-04 | 3.20E-04 | 0.387 | -1.96E-04 | 1.72E-04 | 0.255 |
| rs12611523 | A/G | -5.23E-04 | 5.69E-04 | 0.358 | 4.43E-04 | 3.36E-04 | 0.187 | -4.84E-04 | 2.66E-04 | 0.069 | 3.07E-05 | 1.43E-04 | 0.830 |
| rs4128364 | C/T | 7.22E-04 | 5.98E-04 | 0.227 | -6.74E-04 | 3.53E-04 | 0.056 | -1.90E-04 | 2.80E-04 | 0.496 | -1.70E-04 | 1.50E-04 | 0.259 |
| rs4538155 | T/C | -3.43E-04 | 5.93E-04 | 0.563 | 1.98E-04 | 3.50E-04 | 0.571 | 4.32E-04 | 2.77E-04 | 0.119 | -1.66E-04 | 1.49E-04 | 0.266 |
| rs11885663 | T/C | 7.51E-04 | 6.53E-04 | 0.250 | 3.11E-04 | 3.88E-04 | 0.422 | -2.07E-04 | 3.05E-04 | 0.498 | 3.38E-04 | 1.65E-04 | 0.041 |
| rs10173260 | C/T | 2.70E-04 | 5.78E-04 | 0.641 | -5.19E-04 | 3.42E-04 | 0.128 | -5.89E-05 | 2.70E-04 | 0.827 | -1.78E-04 | 1.46E-04 | 0.222 |
| rs374153 | C/T | -2.28E-04 | 7.75E-04 | 0.768 | 2.99E-04 | 4.58E-04 | 0.514 | -1.10E-04 | 3.62E-04 | 0.761 | 1.83E-04 | 1.95E-04 | 0.350 |
| rs75539574 | C/A | -1.21E-03 | 1.02E-03 | 0.232 | 6.17E-04 | 5.99E-04 | 0.303 | -1.29E-05 | 4.75E-04 | 0.978 | -3.73E-04 | 2.56E-04 | 0.145 |
| rs72804080 | G/A | -9.14E-05 | 8.00E-04 | 0.909 | -8.46E-05 | 4.71E-04 | 0.857 | -2.96E-04 | 3.74E-04 | 0.428 | -1.70E-04 | 2.01E-04 | 0.397 |
| rs62120041 | T/C | -5.85E-04 | 1.14E-03 | 0.609 | -3.28E-04 | 6.73E-04 | 0.626 | 1.58E-04 | 5.34E-04 | 0.767 | -1.88E-04 | 2.87E-04 | 0.512 |
| rs2072727 | T/C | -4.96E-04 | 5.69E-04 | 0.384 | 6.32E-04 | 3.37E-04 | 0.061 | -2.20E-04 | 2.66E-04 | 0.409 | 1.51E-04 | 1.44E-04 | 0.293 |
| rs7644809 | T/C | 1.70E-04 | 5.75E-04 | 0.767 | 4.66E-04 | 3.39E-04 | 0.170 | -1.94E-04 | 2.69E-04 | 0.470 | 1.70E-04 | 1.45E-04 | 0.239 |
| rs13088093 | G/T | -1.19E-03 | 5.99E-04 | 0.047 | -7.95E-04 | 3.55E-04 | 0.025 | -4.00E-04 | 2.80E-04 | 0.153 | -1.04E-05 | 1.51E-04 | 0.945 |
| rs7616632 | T/G | -2.56E-04 | 5.66E-04 | 0.651 | -2.77E-04 | 3.35E-04 | 0.407 | -7.37E-04 | 2.65E-04 | 0.005 | 2.99E-05 | 1.43E-04 | 0.834 |
| rs112230981 | A/G | -9.19E-04 | 1.30E-03 | 0.481 | -9.60E-04 | 7.65E-04 | 0.209 | 4.66E-04 | 6.10E-04 | 0.445 | -4.39E-05 | 3.26E-04 | 0.893 |
| rs17732997 | C/G | 1.39E-04 | 5.71E-04 | 0.808 | 2.07E-04 | 3.38E-04 | 0.539 | -3.63E-05 | 2.67E-04 | 0.892 | 1.81E-04 | 1.44E-04 | 0.210 |
| rs13109404 | T/G | -1.98E-03 | 1.09E-03 | 0.070 | 3.29E-04 | 6.52E-04 | 0.613 | -2.34E-04 | 5.10E-04 | 0.646 | -4.00E-05 | 2.78E-04 | 0.885 |
| rs2192528 | A/G | -1.35E-03 | 5.67E-04 | 0.017 | -3.61E-04 | 3.35E-04 | 0.281 | -1.68E-04 | 2.65E-04 | 0.526 | -2.29E-04 | 1.43E-04 | 0.109 |
| rs17427571 | A/G | -5.45E-04 | 6.08E-04 | 0.370 | -8.06E-04 | 3.59E-04 | 0.025 | -4.18E-04 | 2.84E-04 | 0.141 | -1.98E-04 | 1.53E-04 | 0.196 |
| rs35531607 | C/T | 2.73E-04 | 5.67E-04 | 0.630 | 6.38E-04 | 3.35E-04 | 0.057 | 3.26E-04 | 2.65E-04 | 0.219 | 2.49E-04 | 1.43E-04 | 0.081 |
| rs56372231 | T/C | 6.06E-04 | 5.99E-04 | 0.311 | 3.16E-04 | 3.54E-04 | 0.372 | -3.12E-04 | 2.80E-04 | 0.264 | 3.56E-04 | 1.51E-04 | 0.018 |
| rs180769 | T/C | 1.24E-03 | 5.73E-04 | 0.030 | 4.32E-04 | 3.38E-04 | 0.202 | -1.39E-04 | 2.68E-04 | 0.605 | 1.93E-04 | 1.44E-04 | 0.180 |
| rs11567976 | T/C | 3.05E-04 | 5.71E-04 | 0.594 | 3.34E-04 | 3.37E-04 | 0.321 | -3.74E-05 | 2.67E-04 | 0.889 | -2.42E-04 | 1.43E-04 | 0.092 |
| rs365663 | A/G | -1.53E-04 | 5.68E-04 | 0.787 | 6.94E-06 | 3.36E-04 | 0.984 | -1.05E-05 | 2.66E-04 | 0.969 | 2.89E-04 | 1.43E-04 | 0.044 |
| rs151014368 | A/G | 1.09E-03 | 7.05E-04 | 0.121 | -3.58E-04 | 4.16E-04 | 0.389 | 3.61E-05 | 3.29E-04 | 0.913 | -5.40E-05 | 1.77E-04 | 0.761 |
| rs460692 | C/T | -1.00E-03 | 8.34E-04 | 0.230 | 7.40E-04 | 4.91E-04 | 0.131 | 4.39E-04 | 3.90E-04 | 0.260 | 2.52E-04 | 2.09E-04 | 0.228 |
| rs34556183 | A/G | -6.67E-04 | 6.28E-04 | 0.288 | -3.47E-04 | 3.72E-04 | 0.351 | -1.43E-04 | 2.93E-04 | 0.627 | 4.53E-04 | 1.59E-04 | 0.004 |
| rs80193650 | G/A | 9.16E-05 | 7.65E-04 | 0.905 | 4.65E-04 | 4.53E-04 | 0.305 | 1.59E-04 | 3.58E-04 | 0.656 | -7.92E-05 | 1.93E-04 | 0.682 |
| rs113113059 | T/C | -2.71E-04 | 6.82E-04 | 0.691 | -2.95E-04 | 4.03E-04 | 0.465 | -1.49E-04 | 3.19E-04 | 0.639 | -7.70E-05 | 1.72E-04 | 0.654 |
| rs9382445 | T/C | -4.88E-04 | 5.81E-04 | 0.402 | -2.09E-04 | 3.44E-04 | 0.544 | 3.05E-04 | 2.72E-04 | 0.262 | 2.44E-04 | 1.47E-04 | 0.097 |
| rs2231265 | G/A | -1.43E-04 | 6.73E-04 | 0.831 | 3.73E-04 | 3.97E-04 | 0.349 | 3.15E-04 | 3.15E-04 | 0.316 | -1.45E-05 | 1.69E-04 | 0.932 |
| rs9345234 | C/A | -5.13E-04 | 5.73E-04 | 0.371 | 4.20E-05 | 3.39E-04 | 0.902 | 6.29E-05 | 2.68E-04 | 0.814 | 2.58E-05 | 1.45E-04 | 0.859 |
| rs2079070 | C/G | -9.75E-05 | 6.40E-04 | 0.879 | -2.24E-04 | 3.79E-04 | 0.555 | -2.82E-04 | 2.99E-04 | 0.345 | -1.30E-05 | 1.61E-04 | 0.936 |
| rs7806045 | T/C | 7.57E-05 | 6.55E-04 | 0.908 | 5.09E-04 | 3.87E-04 | 0.188 | -1.49E-04 | 3.06E-04 | 0.626 | 1.23E-04 | 1.65E-04 | 0.457 |
| rs34731055 | T/C | -1.29E-03 | 7.38E-04 | 0.080 | 4.78E-04 | 4.36E-04 | 0.273 | -1.76E-04 | 3.45E-04 | 0.610 | 2.17E-04 | 1.86E-04 | 0.243 |
| rs73219758 | G/A | 8.28E-04 | 6.23E-04 | 0.183 | -1.43E-04 | 3.68E-04 | 0.698 | -4.03E-04 | 2.91E-04 | 0.166 | 2.73E-04 | 1.57E-04 | 0.082 |
| rs330088 | C/T | -2.33E-04 | 5.68E-04 | 0.682 | -3.62E-04 | 3.35E-04 | 0.281 | 9.38E-06 | 2.66E-04 | 0.972 | -8.75E-06 | 1.43E-04 | 0.951 |
| rs1776776 | T/C | 6.24E-07 | 8.50E-04 | 0.999 | 1.08E-04 | 5.04E-04 | 0.830 | 1.74E-04 | 3.98E-04 | 0.662 | -5.12E-05 | 2.15E-04 | 0.811 |
| rs10973207 | T/G | -1.81E-05 | 7.79E-04 | 0.981 | -1.08E-04 | 5.04E-04 | 0.830 | -1.40E-04 | 3.64E-04 | 0.701 | 1.04E-04 | 1.97E-04 | 0.598 |

SNP=single nucleotide polymorphism; E/A=effect/alternative alleles; EAF=effect allele frequency; Beta = per allele effect on SD units; SE = standard error; P value = p-value for the genetic association.

Table 8. The information of long sleep duration instrument SNPs with AF and HF in single sex

| SNP | Alleles  (E/A) | AF in male | | | AF in female | | | HF in male | | | HF in female | | |
| --- | --- | --- | --- | --- | --- | --- | --- | --- | --- | --- | --- | --- | --- |
|  |  | Beta | SE | P value | Beta | SE | P value | Beta | SE | P value | Beta | SE | P value |
| rs6737318 | A/G | 9.41E-04 | 6.85E-04 | 0.170 | -4.68E-04 | 4.04E-04 | 0.247 | 2.69E-04 | 3.20E-04 | 0.401 | 1.96E-04 | 1.72E-04 | 0.254 |
| rs75458655 | C/T | -2.07E-03 | 1.91E-03 | 0.279 | -1.50E-03 | 1.12E-03 | 0.179 | -5.92E-04 | 8.94E-04 | 0.508 | -9.86E-04 | 4.77E-04 | 0.039 |
| rs17688916 | T/A | -2.20E-03 | 7.13E-04 | 0.002 | -3.63E-04 | 4.21E-04 | 0.388 | 2.06E-04 | 3.33E-04 | 0.537 | 7.59E-05 | 1.79E-04 | 0.672 |
| rs17817288 | A/G | 6.25E-04 | 5.66E-04 | 0.270 | -3.12E-04 | 3.33E-04 | 0.349 | -1.73E-04 | 2.65E-04 | 0.514 | -1.37E-04 | 1.42E-04 | 0.335 |
| rs549961083 | C/T | 5.34E-03 | 7.92E-03 | 0.500 | -7.02E-04 | 4.58E-03 | 0.878 | 1.88E-03 | 3.70E-03 | 0.611 | 5.15E-04 | 1.95E-03 | 0.792 |
| rs3751046 | A/G | -4.72E-04 | 8.00E-04 | 0.555 | -3.97E-04 | 4.73E-04 | 0.402 | 1.69E-04 | 3.74E-04 | 0.651 | 1.80E-04 | 2.02E-04 | 0.373 |
| rs7534398 | T/A | -2.54E-04 | 7.08E-04 | 0.720 | 2.32E-04 | 4.19E-04 | 0.579 | 8.35E-05 | 3.31E-04 | 0.801 | 2.32E-04 | 1.79E-04 | 0.194 |

SNP=single nucleotide polymorphism; E/A=effect/alternative alleles; EAF=effect allele frequency; Beta = per allele effect on SD units; SE = standard error; P value = p-value for the genetic association.

Table 9. The information of short sleep duration instrument SNPs with AF and HF in single sex

| SNP | Alleles  (E/A) | AF in male | | | AF in female | | | HF in male | | | HF in female | | | | | |
| --- | --- | --- | --- | --- | --- | --- | --- | --- | --- | --- | --- | --- | --- | --- | --- | --- |
|  |  | Beta | SE | P value | Beta | SE | P value | Beta | SE | P value | | Beta | SE | P value | |  |
| rs2820313 | A/G | -4.94E-04 | 5.96E-04 | 0.406 | -2.09E-04 | 3.51E-04 | 0.551 | -4.50E-04 | 2.78E-04 | 0.106 | -2.85E-04 | | 1.50E-04 | | 0.057 |  |
| rs7524118 | T/C | -2.36E-04 | 6.20E-04 | 0.704 | -1.43E-04 | 3.67E-04 | 0.697 | -4.13E-04 | 2.90E-04 | 0.154 | 2.06E-04 | | 1.56E-04 | | 0.188 |  |
| rs2186122 | A/T | -1.49E-04 | 5.72E-04 | 0.795 | -1.11E-04 | 3.39E-04 | 0.743 | -6.24E-05 | 2.67E-04 | 0.816 | -2.09E-04 | | 1.45E-04 | | 0.149 |  |
| rs12567114 | G/A | -8.68E-04 | 6.35E-04 | 0.171 | -2.22E-04 | 3.75E-04 | 0.554 | -9.09E-05 | 2.97E-04 | 0.759 | 1.61E-04 | | 1.60E-04 | | 0.315 |  |
| rs1607227 | G/T | 5.69E-04 | 6.20E-04 | 0.359 | -1.44E-04 | 3.67E-04 | 0.694 | 4.62E-04 | 2.90E-04 | 0.111 | -1.01E-04 | | 1.56E-04 | | 0.518 |  |
| rs7939345 | T/G | -8.42E-04 | 6.97E-04 | 0.227 | 4.97E-04 | 4.12E-04 | 0.227 | -2.42E-04 | 3.26E-04 | 0.458 | -1.80E-04 | | 1.75E-04 | | 0.306 |  |
| rs17388803 | A/C | 8.58E-04 | 9.42E-04 | 0.362 | 1.57E-04 | 5.54E-04 | 0.777 | 7.26E-04 | 4.40E-04 | 0.099 | 2.45E-05 | | 2.36E-04 | | 0.917 |  |
| rs59779556 | T/G | -2.42E-04 | 5.69E-04 | 0.670 | -1.27E-04 | 3.36E-04 | 0.706 | -3.15E-04 | 2.66E-04 | 0.236 | 3.97E-05 | | 1.43E-04 | | 0.781 |  |
| rs205024 | C/T | 3.86E-04 | 5.81E-04 | 0.507 | -1.58E-05 | 3.43E-04 | 0.963 | 4.30E-06 | 2.71E-04 | 0.987 | 1.78E-04 | | 1.46E-04 | | 0.223 |  |
| rs12963463 | C/T | -5.28E-04 | 6.25E-04 | 0.398 | 4.77E-06 | 3.69E-04 | 0.990 | 7.71E-05 | 2.92E-04 | 0.792 | 3.44E-05 | | 1.57E-04 | | 0.827 |  |
| rs2863957 | C/A | 9.31E-04 | 6.83E-04 | 0.173 | -4.48E-04 | 4.03E-04 | 0.267 | 2.73E-04 | 3.19E-04 | 0.393 | 2.12E-04 | | 1.72E-04 | | 0.217 |  |
| rs1380703 | A/G | 3.44E-05 | 5.94E-04 | 0.954 | -5.12E-04 | 3.50E-04 | 0.144 | 2.03E-04 | 2.78E-04 | 0.465 | -5.59E-05 | | 1.49E-04 | | 0.708 |  |
| rs75539574 | A/C | 1.21E-03 | 1.02E-03 | 0.232 | -6.17E-04 | 5.99E-04 | 0.303 | 1.29E-05 | 4.75E-04 | 0.978 | 3.73E-04 | | 2.56E-04 | | 0.145 |  |
| rs5757675 | G/T | 9.26E-04 | 6.47E-04 | 0.153 | -7.56E-05 | 3.83E-04 | 0.844 | 1.62E-04 | 3.02E-04 | 0.593 | -2.58E-04 | | 1.63E-04 | | 0.114 |  |
| rs2014830 | C/T | 1.35E-03 | 6.19E-04 | 0.029 | 2.48E-04 | 3.66E-04 | 0.498 | 6.70E-04 | 2.89E-04 | 0.020 | 5.36E-04 | | 1.56E-04 | | 0.001 |  |
| rs13107325 | C/T | -1.69E-03 | 1.07E-03 | 0.115 | 4.17E-05 | 6.38E-04 | 0.948 | -2.11E-04 | 4.99E-04 | 0.673 | -7.67E-05 | | 2.72E-04 | | 0.778 |  |
| rs17005118 | G/A | -6.98E-04 | 6.40E-04 | 0.275 | -5.11E-04 | 3.79E-04 | 0.178 | -3.35E-04 | 2.99E-04 | 0.263 | -1.17E-04 | | 1.61E-04 | | 0.469 |  |
| rs3776864 | A/C | -5.49E-04 | 6.00E-04 | 0.360 | -3.02E-04 | 3.55E-04 | 0.394 | 3.33E-04 | 2.80E-04 | 0.235 | -3.53E-04 | | 1.51E-04 | | 0.020 |  |
| rs4585442 | G/A | 2.22E-04 | 6.11E-04 | 0.716 | 1.27E-04 | 3.61E-04 | 0.726 | -1.38E-04 | 2.86E-04 | 0.628 | 2.33E-04 | | 1.54E-04 | | 0.130 |  |
| rs12518468 | T/C | -1.21E-04 | 6.02E-04 | 0.840 | 3.62E-04 | 3.55E-04 | 0.308 | 4.03E-04 | 2.81E-04 | 0.152 | 1.24E-04 | | 1.51E-04 | | 0.411 |  |
| rs9321171 | C/T | -5.35E-05 | 5.69E-04 | 0.925 | 5.92E-04 | 3.36E-04 | 0.078 | 1.92E-04 | 2.66E-04 | 0.470 | -1.56E-04 | | 1.43E-04 | | 0.278 |  |
| rs142180737 | T/C | -3.40E-03 | 3.10E-03 | 0.273 | 2.33E-04 | 1.84E-03 | 0.899 | -3.24E-03 | 1.45E-03 | 0.026 | -9.60E-04 | | 7.83E-04 | | 0.220 |  |
| rs12661667 | C/T | -3.21E-05 | 6.40E-04 | 0.960 | -2.65E-04 | 3.79E-04 | 0.484 | 5.27E-05 | 2.99E-04 | 0.860 | -4.72E-05 | | 1.61E-04 | | 0.770 |  |
| rs9367621 | T/A | 4.50E-04 | 5.71E-04 | 0.431 | 2.14E-04 | 3.38E-04 | 0.526 | -4.11E-04 | 2.67E-04 | 0.123 | -1.05E-04 | | 1.44E-04 | | 0.467 |  |
| rs1229762 | C/T | 1.18E-04 | 5.99E-04 | 0.843 | 2.86E-05 | 3.54E-04 | 0.936 | -1.44E-04 | 2.80E-04 | 0.606 | -6.38E-05 | | 1.51E-04 | | 0.673 |  |
| rs11763750 | G/A | 1.45E-03 | 7.29E-04 | 0.047 | -3.69E-04 | 4.30E-04 | 0.391 | 2.24E-04 | 3.41E-04 | 0.511 | -1.73E-04 | | 1.84E-04 | | 0.346 |  |
| rs60882754 | A/T | 1.80E-03 | 1.18E-03 | 0.126 | 4.99E-05 | 6.95E-04 | 0.943 | -1.41E-04 | 5.50E-04 | 0.797 | -3.99E-04 | | 2.96E-04 | | 0.178 |  |

SNP=single nucleotide polymorphism; E/A=effect/alternative alleles; EAF=effect allele frequency; Beta = per allele effect on SD units; SE = standard error; P value = p-value for the genetic association.

Table 10. Calculation of linkage disequilibrium of selected SNPs of continuous sleep duration

| RS_number | rs915416 | rs269054 | rs61796569 | rs12567114 |
| --- | --- | --- | --- | --- |
| rs915416 | 1.0 | 0.0 | 0.005 | 0.0 |
| rs269054 | 0.0 | 1.0 | 0.0 | 0.005 |
| rs61796569 | 0.005 | 0.0 | 1.0 | 0.001 |
| rs12567114 | 0.0 | 0.005 | 0.001 | 1.0 |

The 4 SNPs in the table are on chromosome 1.

| RS_number | rs12246842 | rs10761674 | rs11190970 | rs7915425 |
| --- | --- | --- | --- | --- |
| rs12246842 | 1.0 | 0.003 | 0.012 | 0.003 |
| rs10761674 | 0.003 | 1.0 | 0.001 | 0.002 |
| rs11190970 | 0.012 | 0.001 | 1.0 | 0.008 |
| rs7915425 | 0.003 | 0.002 | 0.008 | 1.0 |

The 4 SNPs in the table are on chromosome 10.

| RS_number | rs1517572 | rs4592416 | rs11602180 | rs174560 | rs12791153 | rs1553132 | rs1939455 | rs7115226 | rs1263056 | rs7951019 | rs1057703 |
| --- | --- | --- | --- | --- | --- | --- | --- | --- | --- | --- | --- |
| rs1517572 | 1.0 | 0.001 | 0.0 | 0.002 | 0.0 | 0.0 | 0.01 | 0.038 | 0.0 | 0.001 | 0.006 |
| rs4592416 | 0.001 | 1.0 | 0.019 | 0.001 | 0.002 | 0.001 | 0.002 | 0.012 | 0.014 | 0.01 | 0.007 |
| rs11602180 | 0.0 | 0.019 | 1.0 | 0.0 | 0.002 | 0.001 | 0.004 | 0.001 | 0.0 | 0.002 | 0.007 |
| rs174560 | 0.002 | 0.001 | 0.0 | 1.0 | 0.008 | 0.0 | 0.0 | 0.001 | 0.005 | 0.0 | 0.022 |
| rs12791153 | 0.0 | 0.002 | 0.002 | 0.008 | 1.0 | 0.0 | 0.01 | 0.004 | 0.002 | 0.005 | 0.001 |
| rs1553132 | 0.0 | 0.001 | 0.001 | 0.0 | 0.0 | 1.0 | 0.014 | 0.001 | 0.0 | 0.005 | 0.01 |
| rs1939455 | 0.01 | 0.002 | 0.004 | 0.0 | 0.01 | 0.014 | 1.0 | 0.005 | 0.004 | 0.01 | 0.0 |
| rs7115226 | 0.038 | 0.012 | 0.001 | 0.001 | 0.004 | 0.001 | 0.005 | 1.0 | 0.0 | 0.001 | 0.006 |
| rs1263056 | 0.0 | 0.014 | 0.0 | 0.005 | 0.002 | 0.0 | 0.004 | 0.0 | 1.0 | 0.002 | 0.001 |
| rs7951019 | 0.001 | 0.01 | 0.002 | 0.0 | 0.005 | 0.005 | 0.01 | 0.001 | 0.002 | 1.0 | 0.001 |
| rs1057703 | 0.006 | 0.007 | 0.007 | 0.022 | 0.001 | 0.01 | 0.0 | 0.006 | 0.001 | 0.001 | 1.0 |

The 11 SNPs in the table are on chromosome 11.

| RS_number | rs34354917 | rs11614986 | rs4767550 |
| --- | --- | --- | --- |
| rs34354917 | 1.0 | 0.0 | 0.004 |
| rs11614986 | 0.0 | 1.0 | 0.004 |
| rs4767550 | 0.004 | 0.004 | 1.0 |

The 3 SNPs in the table are on chromosome 12.

| RS_number | rs6575005 | rs10483350 | rs61985058 | rs55658675 | rs11621908 |
| --- | --- | --- | --- | --- | --- |
| rs6575005 | 1.0 | 0.002 | 0.005 | 0.002 | 0.0 |
| rs10483350 | 0.002 | 1.0 | 0.002 | 0.008 | 0.008 |
| rs61985058 | 0.005 | 0.002 | 1.0 | 0.017 | 0.0 |
| rs55658675 | 0.002 | 0.008 | 0.017 | 1.0 | 0.001 |
| rs11621908 | 0.0 | 0.008 | 0.0 | 0.001 | 1.0 |

The 5 SNPs in the table are on chromosome 14.

| RS_number | rs3095508 | rs11643715 | rs9940646 | rs8050478 |
| --- | --- | --- | --- | --- |
| rs3095508 | 1.0 | 0.005 | 0.001 | 0.001 |
| rs11643715 | 0.005 | 1.0 | 0.001 | 0.001 |
| rs9940646 | 0.001 | 0.001 | 1.0 | 0.0 |
| rs8050478 | 0.001 | 0.001 | 0.0 | 1.0 |

The 4 SNPs in the table are on chromosome 16.

| RS_number | rs7503199 | rs205024 | rs1991556 | rs9903973 |
| --- | --- | --- | --- | --- |
| rs7503199 | 1.0 | 0.004 | 0.007 | 0.001 |
| rs205024 | 0.004 | 1.0 | 0.009 | 0.003 |
| rs1991556 | 0.007 | 0.009 | 1.0 | 0.001 |
| rs9903973 | 0.001 | 0.003 | 0.001 | 1.0 |

The 4 SNPs in the table are on chromosome 17.

| RS_number | rs62120041 | rs374153 | rs75539574 | rs72804080 | rs7556815 | rs12611523 | rs4128364 | rs4538155 | rs11885663 | rs10173260 |
| --- | --- | --- | --- | --- | --- | --- | --- | --- | --- | --- |
| rs62120041 | 1.0 | 0.002 | 0.001 | 0.014 | 0.003 | 0.001 | 0.006 | 0.009 | 0.001 | 0.001 |
| rs374153 | 0.002 | 1.0 | 0.025 | 0.003 | 0.009 | 0.001 | 0.017 | 0.01 | 0.0 | 0.003 |
| rs75539574 | 0.001 | 0.025 | 1.0 | 0.03 | 0.0 | 0.0 | 0.012 | 0.0 | 0.002 | 0.004 |
| rs72804080 | 0.014 | 0.003 | 0.03 | 1.0 | 0.0 | 0.029 | 0.002 | 0.009 | 0.0 | 0.018 |
| rs7556815 | 0.003 | 0.009 | 0.0 | 0.0 | 1.0 | 0.0 | 0.01 | 0.0 | 0.0 | 0.005 |
| rs12611523 | 0.001 | 0.001 | 0.0 | 0.029 | 0.0 | 1.0 | 0.0 | 0.002 | 0.004 | 0.002 |
| rs4128364 | 0.006 | 0.017 | 0.012 | 0.002 | 0.01 | 0.0 | 1.0 | 0.003 | 0.003 | 0.0 |
| rs4538155 | 0.009 | 0.01 | 0.0 | 0.009 | 0.0 | 0.002 | 0.003 | 1.0 | 0.014 | 0.012 |
| rs11885663 | 0.001 | 0.0 | 0.002 | 0.0 | 0.0 | 0.004 | 0.003 | 0.014 | 1.0 | 0.011 |
| rs10173260 | 0.001 | 0.003 | 0.004 | 0.018 | 0.005 | 0.002 | 0.0 | 0.012 | 0.011 | 1.0 |

The 10 SNPs in the table are on chromosome 2.

| RS_number | rs112230981 | rs17732997 | rs7644809 | rs13088093 | rs7616632 |
| --- | --- | --- | --- | --- | --- |
| rs112230981 | 1.0 | 0.0 | 0.001 | 0.0 | 0.0 |
| rs17732997 | 0.0 | 1.0 | 0.001 | 0.0 | 0.0 |
| rs7644809 | 0.001 | 0.001 | 1.0 | 0.0 | 0.008 |
| rs13088093 | 0.0 | 0.0 | 0.0 | 1.0 | 0.036 |
| rs7616632 | 0.0 | 0.0 | 0.008 | 0.036 | 1.0 |

The 5 SNPs in the table are on chromosome 3.

| RS_number | rs2192528 | rs17427571 | rs35531607 | rs13109404 |
| --- | --- | --- | --- | --- |
| rs2192528 | 1.0 | 0.001 | 0.0 | 0.003 |
| rs17427571 | 0.001 | 1.0 | 0.003 | 0.004 |
| rs35531607 | 0.0 | 0.003 | 1.0 | 0.002 |
| rs13109404 | 0.003 | 0.004 | 0.002 | 1.0 |

The 4 SNPs in the table are on chromosome 4.

| RS_number | rs365663 | rs460692 | rs56372231 | rs180769 | rs11567976 | rs151014368 |
| --- | --- | --- | --- | --- | --- | --- |
| rs365663 | 1.0 | 0.009 | 0.0 | 0.003 | 0.003 | 0.0 |
| rs460692 | 0.009 | 1.0 | 0.001 | 0.006 | 0.0 | 0.001 |
| rs56372231 | 0.0 | 0.001 | 1.0 | 0.0 | 0.004 | 0.001 |
| rs180769 | 0.003 | 0.006 | 0.0 | 1.0 | 0.004 | 0.003 |
| rs11567976 | 0.003 | 0.0 | 0.004 | 0.004 | 1.0 | 0.017 |
| rs151014368 | 0.0 | 0.001 | 0.001 | 0.003 | 0.017 | 1.0 |

The 6 SNPs in the table are on chromosome 5.

| RS_number | rs34556183 | rs80193650 | rs113113059 | rs9382445 | rs2231265 | rs9345234 |
| --- | --- | --- | --- | --- | --- | --- |
| rs34556183 | 1.0 | 0.0 | 0.001 | 0.002 | 0.008 | 0.002 |
| rs80193650 | 0.0 | 1.0 | 0.002 | 0.004 | 0.001 | 0.01 |
| rs113113059 | 0.001 | 0.002 | 1.0 | 0.002 | 0.01 | 0.004 |
| rs9382445 | 0.002 | 0.004 | 0.002 | 1.0 | 0.0 | 0.007 |
| rs2231265 | 0.008 | 0.001 | 0.01 | 0.0 | 1.0 | 0.005 |
| rs9345234 | 0.002 | 0.01 | 0.004 | 0.007 | 0.005 | 1.0 |

The 6 SNPs in the table are on chromosome 6.

| RS_number | rs34731055 | rs2079070 | rs7806045 |
| --- | --- | --- | --- |
| rs34731055 | 1.0 | 0.001 | 0.0 |
| rs2079070 | 0.001 | 1.0 | 0.0 |
| rs7806045 | 0.0 | 0.0 | 1.0 |

The 3 SNPs in the table are on chromosome 7.

| RS_number | rs330088 | rs73219758 |
| --- | --- | --- |
| rs330088 | 1.0 | 0.0 |
| rs73219758 | 0.0 | 1.0 |

The 2 SNPs in the table are on chromosome 8.

| RS_number | rs10973207 | rs1776776 |
| --- | --- | --- |
| rs10973207 | 1.0 | 0.001 |
| rs1776776 | 0.001 | 1.0 |

The 2 SNPs in the table are on chromosome 9.

Population = (CEU) Utah Residents from North and West Europe; r2 < 0.1.

Table 11. Calculation of linkage disequilibrium of selected SNPs of short sleep duration

| RS number | rs915416 | rs269054 | rs61796569 | rs12567114 |
| --- | --- | --- | --- | --- |
| rs915416 | 1.0 | 0.0 | 0.005 | 0.0 |
| rs269054 | 0.0 | 1.0 | 0.0 | 0.005 |
| rs61796569 | 0.005 | 0.0 | 1.0 | 0.001 |
| rs12567114 | 0.0 | 0.005 | 0.001 | 1.0 |

The 4 SNPs in the table are on chromosome 1.

| RS_number | rs1380703 | rs75539574 | rs2863957 |
| --- | --- | --- | --- |
| rs1380703 | 1.0 | 0.016 | 0.0 |
| rs75539574 | 0.016 | 1.0 | 0.0 |
| rs2863957 | 0.0 | 0.0 | 1.0 |

The 3 SNPs in the table are on chromosome 2.

| RS_number | rs17005118 | rs13107325 |
| --- | --- | --- |
| rs17005118 | 1.0 | 0.009 |
| rs13107325 | 0.009 | 1.0 |

The 2 SNPs in the table are on chromosome 4.

| RS_number | rs12518468 | rs3776864 | rs4585442 |
| --- | --- | --- | --- |
| rs12518468 | 1.0 | 0.0 | 0.0 |
| rs3776864 | 0.0 | 1.0 | 0.0 |
| rs4585442 | 0.0 | 0.0 | 1.0 |

The 3 SNPs in the table are on chromosome 5.

| RS_number | rs142180737 | rs12661667 | rs9367621 | rs9321171 |
| --- | --- | --- | --- | --- |
| rs142180737 | 1.0 | 0.002 | 0.024 | 0.0 |
| rs12661667 | 0.002 | 1.0 | 0.001 | 0.001 |
| rs9367621 | 0.024 | 0.001 | 1.0 | 0.0 |
| rs9321171 | 0.0 | 0.001 | 0.0 | 1.0 |

The 4 SNPs in the table are on chromosome 6.

| RS_number | rs11763750 | rs1229762 |
| --- | --- | --- |
| rs11763750 | 1.0 | 0.002 |
| rs1229762 | 0.002 | 1.0 |

The 4 SNPs in the table are on chromosome 7.

| RS_number | rs1607227 | rs7939345 |
| --- | --- | --- |
| rs1607227 | 1.0 | 0.0 |
| rs7939345 | 0.0 | 1.0 |

The 2 SNPs in the table are on chromosome 11.

Population = (CEU) Utah Residents from North and West Europe; r2 < 0.1.

Table 12. Calculation of linkage disequilibrium of selected SNPs of long sleep duration.

| RS_number | rs10899257 | rs75458655 | rs3751046 |
| --- | --- | --- | --- |
| rs10899257 | 1.0 | 0.004 | 0.016 |
| rs75458655 | 0.004 | 1.0 | 0.0 |
| rs3751046 | 0.016 | 0.0 | 1.0 |

The 3 SNPs in the table are on chromosome 11.

Population = (CEU) Utah Residents from North and West Europe; r2 < 0.1.

Table 13. Sensitivity analysis for genetic associations between sleep duration and AF and HF

| **Sleep Duration on Atrial Fibrillation** | OR (95% CI) | P value |
| --- | --- | --- |
| ***Continuous Sleep Duration*** | | |
| Sensitivity IVW  Weighted median | 0.864 (0.960-0.984)  0.911 (0.769-1.080) | 0.027  0.282 |
| MR-Egger  (intercept) | 0.987 (0.519-1.875) | 0.967  0.751 |
| Male IVW  Female IVW | 0.989 (0.981-0.998)  1.001 (0.996-1.007) | 0.019  0.643 |
| ***Short Sleep Duration*** | | |
| Weighted median | 1.119 (1.041-1.358) | 0.011 |
| MR-Egger  (intercept) | 1.044 (0.661-1.649) | 0.853  0.791 |
| Male IVW  Female IVW | 1.008 (1.001-1.015)  1.000 (0.996-1.005) | 0.031  0.832 |
| ***Long Sleep Duration*** | | |
| Weighted median | 0.923 (0.790-1.078) | 0.312 |
| MR-Egger  (intercept) | 1.222 (0.837-1.784) | 0.300  0.161 |
| Male IVW  Female IVW | 0.995 (0.987-1.003)  1.002 (0.998-1.007) | 0.235  0.316 |
| **Sleep Duration on Heart Failure** | OR (95% CI) | P value |
| ***Continuous Sleep Duration*** |  |  |
| Sensitivity IVW  Weighted median | 0.780 (0.680-0.894)  0.799 (0.653-0.978) | 3.70×10^-4^  0.030 |
| MR-Egger  (intercept) | 1.074 (0.658-1.754) | 0.774  0.159 |
| Male IVW  Female IVW | 0.995 (0.991-0.999)  1.001 (0.999-1.003) | 0.021  0.474 |
| ***Short Sleep Duration*** |  |  |
| Weighted median | 1.066 (0.919-1.238) | 0.398 |
| MR-Egger  (intercept) | 1.444 (0.878-2.376) | 0.148  0.329 |
| Male IVW  Female IVW | 1.003 (0.999-1.006)  1.000 (0.999-1.002) | 0.105  0.641 |
| ***Long Sleep Duration*** |  |  |
| Weighted median | 0.909 (0.774-1.068) | 0.247 |
| MR-Egger  (intercept) | 1.038 (0.674-1.597) | 0.164  0.572 |
| Male IVW  Female IVW | 0.999 (0.996-1.003)  0.999 (0.997-1.001) | 0.705  0.599 |

OR = odds ratio; CI = confidence interval; P value = p-value of the causal estimate.

Table 14. Characteristics of SNPs for atrial fibrillation instrument from GWAS meta-analysis

| SNP | Chromosome position | Nearest Gene | Alleles  (E/A) | EAF | Beta | SE | P value |
| --- | --- | --- | --- | --- | --- | --- | --- |
| rs284277 | 1:10790797 | CASZ1 | C/A | 0.383 | 0.0392 | 0.0066 | 1.2E-09 |
| rs7529220 | 1:22282619 | HSPG2 | C/T | 0.847 | 0.0583 | 0.0093 | 2.0E-10 |
| rs2885697 | 1:41544279 | SCMH1 | G/T | 0.352 | 0.0392 | 0.0063 | 2.9E-10 |
| rs11590635 | 1:49309764 | AGBL4 | A/G | 0.024 | 0.1484 | 0.0258 | 4.1E-09 |
| rs146518726 | 1:51535039 | MIR6500 | A/G | 0.033 | 0.1570 | 0.0205 | 8.3E-15 |
| rs1545300 | 1:112464004 | KCND3 | C/T | 0.691 | 0.0583 | 0.0077 | 1.5E-14 |
| rs4073778 | 1:116297758 | CASQ2 | A/C | 0.564 | 0.0488 | 0.0068 | 5.0E-13 |
| rs79187193 | 1:147255831 | GJA5 | G/A | 0.943 | 0.1133 | 0.0151 | 3.2E-14 |
| rs11264280 | 1:154862952 | KCNN3 | T/C | 0.333 | 0.1310 | 0.0070 | 3.1E-79 |
| rs72700114 | 1:170193825 | LINC01142 | C/G | 0.076 | 0.1989 | 0.0129 | 3.3E-54 |
| rs10753933 | 1:203026214 | PPFIA4 | T/G | 0.448 | 0.0583 | 0.0065 | 9.8E-20 |
| rs4951258 | 1:205691316 | NUCKS1 | A/G | 0.416 | 0.0392 | 0.0072 | 2.1E-08 |
| rs7578393 | 2:26165528 | KIF3C | T/C | 0.796 | 0.0583 | 0.0084 | 2.4E-12 |
| rs11125871 | 2:61470126 | USP34 | C/T | 0.605 | 0.0392 | 0.0069 | 6.4E-09 |
| rs2540949 | 2:65284231 | CEP68 | A/T | 0.615 | 0.0677 | 0.0070 | 2.9E-22 |
| rs6747542 | 2:70106832 | GMCL1 | T/C | 0.536 | 0.0583 | 0.0071 | 1.1E-16 |
| rs72926475 | 2:86594487 | REEP1 | G/A | 0.877 | 0.0677 | 0.0103 | 2.4E-11 |
| rs28387148 | 2:127433465 | GYPC | T/C | 0.105 | 0.0770 | 0.0120 | 6.3E-11 |
| rs67969609 | 2:145760353 | TEX41 | G/C | 0.071 | 0.0677 | 0.0123 | 1.7E-08 |
| rs56181519 | 2:175555714 | WIPF1 | C/T | 0.732 | 0.0677 | 0.0079 | 6.5E-18 |
| rs2288327 | 2:179411665 | TTN | G/A | 0.156 | 0.0953 | 0.0093 | 7.3E-25 |
| rs3820888 | 2:201180023 | SPATS2L | C/T | 0.392 | 0.0677 | 0.0067 | 5.7E-24 |
| rs35544454 | 2:213266003 | ERBB4 | A/T | 0.808 | 0.0583 | 0.0087 | 1.1E-11 |
| rs7650482 | 3:12841804 | CAND2 | G/A | 0.640 | 0.0677 | 0.0067 | 1.8E-24 |
| rs73041705 | 3:24463235 | THRB | T/C | 0.702 | 0.0488 | 0.0082 | 1.5E-09 |
| rs6790396 | 3:38771925 | SCN10A | G/C | 0.596 | 0.0583 | 0.0064 | 2.4E-20 |
| rs34080181 | 3:66454191 | LRIG1 | G/A | 0.621 | 0.0488 | 0.0077 | 1.3E-10 |
| rs17005647 | 3:69406181 | FRMD4B | T/C | 0.364 | 0.0392 | 0.0067 | 2.7E-09 |
| rs6771054 | 3:89489529 | EPHA3 | T/C | 0.596 | 0.0488 | 0.0074 | 2.4E-11 |
| rs10804493 | 3:111554426 | PHLDB2 | A/G | 0.651 | 0.0583 | 0.0074 | 1.6E-15 |
| rs1278493 | 3:135814009 | PPP2R3A | G/A | 0.436 | 0.0392 | 0.0070 | 8.8E-09 |
| rs7612445 | 3:179172979 | GNB4 | T/G | 0.188 | 0.0488 | 0.0085 | 4.8E-09 |
| rs60902112 | 3:194800853 | XXYLT1 | T/C | 0.226 | 0.0488 | 0.0088 | 1.7E-08 |
| rs1458038 | 4:81164723 | FGF5 | T/C | 0.309 | 0.0392 | 0.0066 | 1.7E-09 |
| rs10006327 | 4:103890980 | SLC9B1 | C/T | 0.490 | 0.0392 | 0.0073 | 4.4E-08 |
| rs67249485 | 4:111699685 | PITX2 | T/A | 0.199 | 0.3655 | 0.0081 | 7.3E-443 |
| rs6829664 | 4:114448656 | CAMK2D | G/A | 0.262 | 0.0583 | 0.0080 | 1.9E-13 |
| rs10213171 | 4:148937537 | ARHGAP10 | G/C | 0.061 | 0.0953 | 0.0143 | 1.3E-11 |
| rs12648245 | 4:174641184 | HAND2 | T/C | 0.924 | 0.0953 | 0.0133 | 3.5E-13 |
| rs6596717 | 5:106427609 | LOC102467213 | C/A | 0.395 | 0.0392 | 0.0067 | 3.0E-09 |
| rs337705 | 5:113737062 | KCNN2 | G/T | 0.375 | 0.0583 | 0.0071 | 1.6E-16 |
| rs2012809 | 5:128190363 | SLC27A6 | G/A | 0.790 | 0.0583 | 0.0095 | 4.9E-10 |
| rs2040862 | 5:137419989 | WNT8A | T/C | 0.178 | 0.1044 | 0.0084 | 1.1E-35 |
| rs6580277 | 5:142818123 | NR3C1 | G/A | 0.237 | 0.0677 | 0.0080 | 1.6E-17 |
| rs12188351 | 5:168386089 | SLIT3 | A/G | 0.056 | 0.0862 | 0.0147 | 2.5E-09 |
| rs6891790 | 5:172670745 | NKX2-5 | G/T | 0.717 | 0.0770 | 0.0080 | 4.5E-22 |
| rs73366713 | 6:16415751 | ATXN1 | G/A | 0.860 | 0.1044 | 0.0101 | 1.5E-25 |
| rs34969716 | 6:18210109 | KDM1B | A/G | 0.305 | 0.0677 | 0.0075 | 1.6E-19 |
| rs3176326 | 6:36647289 | CDKN1A | G/A | 0.802 | 0.0583 | 0.0080 | 1.4E-13 |
| rs2031522 | 6:87821501 | CGA | A/G | 0.624 | 0.0392 | 0.0062 | 1.5E-10 |
| rs3951016 | 6:118559658 | SLC35F1 | A/T | 0.459 | 0.0677 | 0.0070 | 2.1E-22 |
| rs13195459 | 6:122403559 | HSF2 | G/A | 0.638 | 0.0583 | 0.0066 | 4.2E-19 |
| rs117984853 | 6:149399100 | UST | T/G | 0.101 | 0.1222 | 0.0120 | 1.3E-24 |
| rs55734480 | 7:14372009 | DGKB | A/G | 0.249 | 0.0583 | 0.0084 | 2.2E-12 |
| rs6462079 | 7:28415827 | CREB5 | A/G | 0.721 | 0.0488 | 0.0081 | 8.8E-10 |
| rs35005436 | 7:74134911 | GTF2I | C/T | 0.155 | 0.0583 | 0.0094 | 3.3E-10 |
| rs56201652 | 7:92278116 | CDK6 | G/A | 0.733 | 0.0488 | 0.0070 | 1.7E-12 |
| rs11773845 | 7:116191301 | CAV1 | A/C | 0.586 | 0.1044 | 0.0067 | 2.4E-55 |
| rs55985730 | 7:128417044 | OPN1SW | G/T | 0.060 | 0.0862 | 0.0151 | 5.2E-09 |
| rs7789146 | 7:150661409 | KCNH2 | G/A | 0.821 | 0.0583 | 0.0088 | 2.1E-11 |
| rs35620480 | 8:11499908 | GATA4 | C/A | 0.157 | 0.0583 | 0.0102 | 5.2E-09 |
| rs7508 | 8:17913970 | ASAH1 | A/G | 0.711 | 0.0677 | 0.0072 | 1.7E-21 |
| rs7834729 | 8:21821778 | XPO7 | G/T | 0.885 | 0.0677 | 0.0110 | 3.6E-10 |
| rs62521286 | 8:124551975 | FBXO32 | G/A | 0.066 | 0.1222 | 0.0138 | 4.5E-19 |
| rs6994744 | 8:141740868 | PTK2 | C/A | 0.495 | 0.0392 | 0.0066 | 1.1E-09 |
| rs10821415 | 9:97713459 | C9orf3 | A/C | 0.413 | 0.0862 | 0.0071 | 2.9E-34 |
| rs2274115 | 9:139094773 | LHX3 | G/A | 0.700 | 0.0488 | 0.0078 | 1.7E-10 |
| rs12245149 | 10:65321147 | REEP3 | C/A | 0.526 | 0.0488 | 0.0070 | 1.7E-12 |
| rs7096385 | 10:69664881 | SIRT1 | T/C | 0.092 | 0.0677 | 0.0127 | 4.9E-08 |
| rs60212594 | 10:75414344 | SYNPO2L | G/C | 0.856 | 0.1133 | 0.0093 | 9.2E-35 |
| rs10458660 | 10:77936576 | C10orf11 | G/A | 0.173 | 0.0583 | 0.0096 | 6.8E-10 |
| rs11598047 | 10:105342672 | NEURL1 | G/A | 0.162 | 0.1570 | 0.0092 | 9.0E-66 |
| rs10749053 | 10:112576695 | RBM20 | T/C | 0.158 | 0.0583 | 0.0104 | 1.0E-08 |
| rs10741807 | 11:20011445 | NAV2 | T/C | 0.245 | 0.0770 | 0.0084 | 1.6E-20 |
| rs4935786 | 11:121661507 | SORL1 | T/A | 0.267 | 0.0488 | 0.0085 | 4.9E-09 |
| rs76097649 | 11:128764570 | KCNJ5 | A/G | 0.093 | 0.1133 | 0.0123 | 1.3E-20 |
| rs4963776 | 12:24779491 | LINC00477 | G/T | 0.818 | 0.0953 | 0.0092 | 1.8E-25 |
| rs17380837 | 12:26345526 | SSPN | C/T | 0.693 | 0.0488 | 0.0072 | 4.8E-12 |
| rs12809354 | 12:32978437 | PKP2 | C/T | 0.144 | 0.0677 | 0.0090 | 2.9E-14 |
| rs2860482 | 12:57105938 | NACA | A/C | 0.274 | 0.0583 | 0.0083 | 1.2E-12 |
| rs71454237 | 12:70013415 | LRRC10 | G/A | 0.791 | 0.0583 | 0.0080 | 1.8E-13 |
| rs12426679 | 12:76237987 | PHLDA1 | C/T | 0.472 | 0.0392 | 0.0068 | 4.9E-09 |
| rs883079 | 12:114793240 | TBX5 | T/C | 0.707 | 0.0953 | 0.0072 | 2.8E-40 |
| rs10773657 | 12:123327900 | HIP1R | C/A | 0.138 | 0.0583 | 0.0107 | 2.5E-08 |
| rs6560886 | 12:133150210 | FBRSL1 | C/T | 0.788 | 0.0488 | 0.0088 | 1.5E-08 |
| rs9506925 | 13:23368943 | LINC00540 | T/C | 0.267 | 0.0488 | 0.0084 | 2.7E-09 |
| rs35569628 | 13:113872712 | CUL4A | T/C | 0.777 | 0.0488 | 0.0088 | 1.4E-08 |
| rs422068 | 14:23864804 | MYH6 | C/T | 0.349 | 0.0392 | 0.0064 | 3.9E-10 |
| rs11156751 | 14:32990437 | AKAP6 | C/T | 0.285 | 0.0677 | 0.0073 | 6.9E-21 |
| rs73241997 | 14:35173775 | CFL2 | T/C | 0.142 | 0.0770 | 0.0099 | 2.9E-15 |
| rs2738413 | 14:64679960 | SYNE2 | A/G | 0.495 | 0.0770 | 0.0066 | 2.5E-31 |
| rs74884082 | 14:73249419 | DPF3 | C/T | 0.750 | 0.0488 | 0.0079 | 3.5E-10 |
| rs10873298 | 14:77426525 | IRF2BPL | C/T | 0.366 | 0.0392 | 0.0069 | 7.1E-09 |
| rs147301839 | 15:57924714 | GCOM1 | C/A | 0.007 | 0.3293 | 0.0526 | 1.9E-10 |
| rs7170477 | 15:64103777 | HERC1 | A/G | 0.304 | 0.0392 | 0.0074 | 5.0E-08 |
| rs74022964 | 15:73677264 | HCN4 | T/C | 0.157 | 0.1133 | 0.0091 | 3.5E-36 |
| rs12908004 | 15:80676925 | ARNT2 | G/A | 0.164 | 0.0770 | 0.0096 | 4.1E-16 |
| rs4965430 | 15:99268850 | IGF1R | C/G | 0.386 | 0.0488 | 0.0077 | 1.3E-10 |
| rs140185678 | 16:2003016 | RPL3L | A/G | 0.035 | 0.1655 | 0.0220 | 2.4E-14 |
| rs2359171 | 16:73053022 | ZFHX3 | A/T | 0.176 | 0.1740 | 0.0086 | 4.7E-91 |
| rs7225165 | 17:1309850 | YWHAE | G/A | 0.887 | 0.0677 | 0.0117 | 3.2E-09 |
| rs9899183 | 17:7452977 | TNFSF12 | T/C | 0.714 | 0.0488 | 0.0083 | 2.0E-09 |
| rs72811294 | 17:12618680 | MYOCD | G/C | 0.887 | 0.0677 | 0.0101 | 9.7E-12 |
| rs11658278 | 17:38031164 | ZPBP2 | T/C | 0.479 | 0.0488 | 0.0075 | 3.5E-11 |
| rs1563304 | 17:44874453 | WNT3 | T/C | 0.178 | 0.0677 | 0.0098 | 2.6E-12 |
| rs12604076 | 17:76773638 | CYTH1 | T/C | 0.478 | 0.0392 | 0.0073 | 3.6E-08 |
| rs9953366 | 18:46474192 | SMAD7 | C/T | 0.663 | 0.0488 | 0.0074 | 1.8E-11 |
| rs8088085 | 18:48708548 | MEX3C | A/C | 0.535 | 0.0392 | 0.0074 | 4.8E-08 |
| rs2834618 | 21:36119111 | LINC01426 | T/G | 0.894 | 0.0953 | 0.0114 | 3.4E-17 |
| rs464901 | 22:18597502 | TUBA8 | T/C | 0.665 | 0.0488 | 0.0070 | 1.5E-12 |
| rs133902 | 22:26164079 | MYO18B | T/C | 0.427 | 0.0392 | 0.0065 | 9.1E-10 |

SNP=single nucleotide polymorphism; E/A=effect/alternative alleles; EAF=effect allele frequency; Beta = per allele effect on SD units; SE = standard error; P value = p-value for the genetic association.

Table 15. The raw information of atrial fibrillation SNPs with sleep duration

| SNP | Alleles  (E/A) | Continuous Sleep Duration | | | Short sleep duration | | | Long sleep duration | | |
| --- | --- | --- | --- | --- | --- | --- | --- | --- | --- | --- |
|  |  | Beta | SE | P value | Beta | SE | P value | Beta | SE | P value |
| rs284277 | C/A | -0.0021 | 0.0024 | 0.35 | 0.0005 | 0.0010 | 0.58 | -0.0006 | 0.0008 | 0.49 |
| rs7529220 | C/T | 0.0061 | 0.0033 | 0.06 | -0.0012 | 0.0014 | 0.37 | 0.0017 | 0.0011 | 0.11 |
| rs2885697 | G/T | 0.0027 | 0.0024 | 0.23 | -0.0002 | 0.0010 | 0.83 | 0.0007 | 0.0008 | 0.35 |
| rs11590635 | A/G | -0.0073 | 0.0078 | 0.38 | 0.0016 | 0.0033 | 0.68 | -0.0009 | 0.0025 | 0.75 |
| rs146518726 | A/G | 0.0046 | 0.0080 | 0.57 | -0.0022 | 0.0034 | 0.50 | 0.0010 | 0.0026 | 0.69 |
| rs1545300 | C/T | 0.0042 | 0.0025 | 0.08 | -0.0013 | 0.0010 | 0.22 | 0.0006 | 0.0008 | 0.42 |
| rs4073778 | A/C | -0.0020 | 0.0023 | 0.44 | 0.0004 | 0.0010 | 0.70 | 0.0005 | 0.0007 | 0.51 |
| rs79187193 | G/A | 0.0106 | 0.0054 | 0.05 | -0.0014 | 0.0023 | 0.60 | 0.0024 | 0.0017 | 0.17 |
| rs11264280 | T/C | -0.0046 | 0.0024 | 0.06 | 0.0012 | 0.0010 | 0.24 | -0.0015 | 0.0008 | 0.06 |
| rs72700114 | C/G | 0.0053 | 0.0044 | 0.23 | -0.0005 | 0.0019 | 0.79 | 0.0020 | 0.0014 | 0.16 |
| rs10753933 | T/G | -0.0023 | 0.0023 | 0.33 | 0.0004 | 0.0010 | 0.71 | 0.0000 | 0.0007 | 0.99 |
| rs4951258 | A/G | 0.0078 | 0.0023 | 0.00 | -0.0031 | 0.0010 | 0.00 | 0.0006 | 0.0007 | 0.45 |
| rs7578393 | T/C | 0.0055 | 0.0030 | 0.06 | -0.0007 | 0.0013 | 0.59 | 0.0020 | 0.0010 | 0.04 |
| rs11125871 | C/T | -0.0034 | 0.0023 | 0.15 | 0.0005 | 0.0010 | 0.58 | -0.0011 | 0.0007 | 0.14 |
| rs2540949 | A/T | -0.0064 | 0.0023 | 0.01 | 0.0033 | 0.0010 | 1.5E-3 | 0.0002 | 0.0007 | 0.81 |
| rs6747542 | T/C | -0.0048 | 0.0023 | 0.03 | 0.0003 | 0.0010 | 0.73 | -0.0010 | 0.0007 | 0.15 |
| rs72926475 | G/A | 0.0012 | 0.0034 | 0.77 | -0.0010 | 0.0015 | 0.51 | -0.0002 | 0.0011 | 0.88 |
| rs28387148 | T/C | 0.0002 | 0.0038 | 0.95 | 0.0007 | 0.0016 | 0.66 | 0.0018 | 0.0012 | 0.13 |
| rs67969609 | G/C | 0.0010 | 0.0045 | 0.85 | 0.0007 | 0.0019 | 0.74 | 0.0020 | 0.0014 | 0.16 |
| rs56181519 | C/T | -0.0027 | 0.0026 | 0.33 | -0.0002 | 0.0011 | 0.78 | -0.0016 | 0.0008 | 0.05 |
| rs2288327 | G/A | -0.0037 | 0.0031 | 0.21 | -0.0004 | 0.0013 | 0.79 | -0.0004 | 0.0010 | 0.65 |
| rs3820888 | C/T | 0.0062 | 0.0023 | 0.01 | -0.0029 | 0.0010 | 3.4E-3 | 0.0001 | 0.0007 | 0.91 |
| rs35544454 | A/T | 0.0025 | 0.0029 | 0.39 | -0.0005 | 0.0012 | 0.70 | 0.0007 | 0.0009 | 0.47 |
| rs7650482 | G/A | -0.0042 | 0.0024 | 0.09 | 0.0011 | 0.0010 | 0.28 | -0.0011 | 0.0008 | 0.15 |
| rs73041705 | T/C | 0.0015 | 0.0025 | 0.57 | 0.0000 | 0.0010 | 1.00 | -0.0003 | 0.0008 | 0.67 |
| rs6790396 | G/C | -0.0037 | 0.0023 | 0.10 | 0.0007 | 0.0010 | 0.45 | -0.0005 | 0.0007 | 0.54 |
| rs34080181 | G/A | 0.0030 | 0.0023 | 0.20 | -0.0004 | 0.0010 | 0.70 | 0.0003 | 0.0007 | 0.65 |
| rs17005647 | T/C | -0.0066 | 0.0024 | 0.01 | 0.0008 | 0.0010 | 0.44 | -0.0010 | 0.0008 | 0.17 |
| rs6771054 | T/C | -0.0003 | 0.0023 | 0.90 | 0.0008 | 0.0010 | 0.44 | 0.0000 | 0.0007 | 1.00 |
| rs10804493 | A/G | -0.0019 | 0.0024 | 0.39 | 0.0006 | 0.0010 | 0.56 | 0.0006 | 0.0008 | 0.43 |
| rs1278493 | G/A | -0.0071 | 0.0023 | 0.00 | 0.0025 | 0.0010 | 0.01 | -0.0006 | 0.0007 | 0.40 |
| rs7612445 | T/G | 0.0041 | 0.0029 | 0.15 | -0.0024 | 0.0012 | 0.05 | -0.0005 | 0.0009 | 0.59 |
| rs60902112 | T/C | 0.0051 | 0.0027 | 0.06 | -0.0004 | 0.0012 | 0.72 | 0.0011 | 0.0009 | 0.21 |
| rs1458038 | T/C | -0.0052 | 0.0025 | 0.04 | 0.0018 | 0.0011 | 0.10 | 0.0003 | 0.0008 | 0.71 |
| rs10006327 | C/T | NA | NA | NA | NA | NA | NA | NA | NA | NA |
| rs67249485 | T/A | -0.0001 | 0.0029 | 0.99 | -0.0008 | 0.0012 | 0.48 | -0.0004 | 0.0009 | 0.64 |
| rs6829664 | G/A | 0.0080 | 0.0026 | 0.00 | -0.0020 | 0.0011 | 0.06 | 0.0011 | 0.0008 | 0.19 |
| rs10213171 | G/C | -0.0070 | 0.0050 | 0.17 | -0.0002 | 0.0021 | 0.91 | -0.0029 | 0.0016 | 0.06 |
| rs12648245 | T/C | NA | NA | NA | NA | NA | NA | NA | NA | NA |
| rs6596717 | C/A | 0.0013 | 0.0023 | 0.58 | 0.0012 | 0.0010 | 0.22 | 0.0014 | 0.0007 | 0.06 |
| rs337705 | G/T | -0.0040 | 0.0023 | 0.10 | 0.0017 | 0.0010 | 0.09 | -0.0006 | 0.0007 | 0.45 |
| rs2012809 | G/A | -0.0025 | 0.0029 | 0.33 | -0.0006 | 0.0012 | 0.64 | -0.0022 | 0.0009 | 0.02 |
| rs2040862 | T/C | -0.0077 | 0.0029 | 0.01 | -0.0002 | 0.0013 | 0.91 | -0.0026 | 0.0009 | 0.01 |
| rs6580277 | G/A | 0.0023 | 0.0026 | 0.37 | -0.0003 | 0.0011 | 0.77 | 0.0001 | 0.0008 | 0.89 |
| rs12188351 | A/G | 0.0001 | 0.0052 | 0.98 | 0.0030 | 0.0022 | 0.17 | 0.0008 | 0.0017 | 0.65 |
| rs6891790 | G/T | -0.0006 | 0.0025 | 0.78 | -0.0002 | 0.0011 | 0.87 | 0.0000 | 0.0008 | 0.95 |
| rs73366713 | G/A | -0.0023 | 0.0032 | 0.46 | 0.0010 | 0.0014 | 0.46 | -0.0005 | 0.0010 | 0.65 |
| rs34969716 | A/G | 0.0016 | 0.0025 | 0.51 | -0.0001 | 0.0011 | 0.96 | -0.0006 | 0.0008 | 0.51 |
| rs3176326 | G/A | 0.0039 | 0.0029 | 0.17 | -0.0032 | 0.0012 | 0.01 | -0.0003 | 0.0009 | 0.73 |
| rs2031522 | A/G | -0.0061 | 0.0023 | 0.01 | 0.0019 | 0.0010 | 0.06 | 0.0001 | 0.0007 | 0.88 |
| rs3951016 | A/T | 0.0073 | 0.0023 | 0.00 | -0.0011 | 0.0010 | 0.26 | 0.0011 | 0.0007 | 0.12 |
| rs13195459 | G/A | 0.0037 | 0.0024 | 0.12 | -0.0014 | 0.0010 | 0.16 | 0.0000 | 0.0008 | 0.98 |
| rs117984853 | T/G | -0.0043 | 0.0039 | 0.27 | -0.0007 | 0.0017 | 0.71 | -0.0019 | 0.0013 | 0.14 |
| rs55734480 | A/G | 0.0068 | 0.0026 | 0.01 | -0.0024 | 0.0011 | 0.03 | 0.0001 | 0.0008 | 0.86 |
| rs6462079 | A/G | 0.0004 | 0.0026 | 0.84 | 0.0004 | 0.0011 | 0.73 | -0.0001 | 0.0008 | 0.92 |
| rs35005436 | C/T | 0.0082 | 0.0031 | 0.01 | -0.0030 | 0.0013 | 0.02 | 0.0009 | 0.0010 | 0.33 |
| rs56201652 | G/A | 0.0017 | 0.0026 | 0.48 | -0.0006 | 0.0011 | 0.58 | -0.0006 | 0.0008 | 0.44 |
| rs11773845 | A/C | -0.0025 | 0.0023 | 0.25 | 0.0013 | 0.0010 | 0.18 | -0.0011 | 0.0007 | 0.13 |
| rs55985730 | G/T | 0.0108 | 0.0050 | 0.03 | -0.0020 | 0.0021 | 0.36 | 0.0016 | 0.0016 | 0.33 |
| rs7789146 | G/A | 0.0013 | 0.0030 | 0.70 | -0.0020 | 0.0013 | 0.12 | -0.0016 | 0.0010 | 0.10 |
| rs35620480 | C/A | 0.0030 | 0.0031 | 0.33 | 0.0004 | 0.0013 | 0.79 | 0.0003 | 0.0010 | 0.75 |
| rs7508 | A/G | -0.0038 | 0.0025 | 0.12 | 0.0002 | 0.0011 | 0.85 | -0.0012 | 0.0008 | 0.15 |
| rs7834729 | G/T | -0.0034 | 0.0036 | 0.32 | -0.0011 | 0.0015 | 0.48 | -0.0032 | 0.0011 | 0.01 |
| rs62521286 | G/A | -0.0041 | 0.0045 | 0.41 | -0.0019 | 0.0019 | 0.31 | -0.0037 | 0.0015 | 0.01 |
| rs6994744 | C/A | -0.0024 | 0.0023 | 0.29 | 0.0016 | 0.0010 | 0.10 | 0.0006 | 0.0007 | 0.39 |
| rs10821415 | A/C | -0.0012 | 0.0023 | 0.59 | 0.0007 | 0.0010 | 0.46 | -0.0005 | 0.0007 | 0.49 |
| rs2274115 | G/A | -0.0053 | 0.0025 | 0.03 | 0.0013 | 0.0011 | 0.24 | -0.0006 | 0.0008 | 0.45 |
| rs12245149 | C/A | -0.0045 | 0.0023 | 0.05 | -0.0016 | 0.0010 | 0.09 | -0.0032 | 0.0007 | 0.00 |
| rs7096385 | T/C | 0.0044 | 0.0046 | 0.35 | -0.0002 | 0.0019 | 0.91 | 0.0014 | 0.0015 | 0.33 |
| rs60212594 | G/C | -0.0043 | 0.0032 | 0.20 | -0.0012 | 0.0014 | 0.36 | -0.0022 | 0.0010 | 0.03 |
| rs10458660 | G/A | 0.0043 | 0.0030 | 0.16 | -0.0019 | 0.0013 | 0.14 | 0.0005 | 0.0010 | 0.59 |
| rs11598047 | G/A | -0.0005 | 0.0032 | 0.88 | 0.0013 | 0.0014 | 0.33 | 0.0001 | 0.0010 | 0.89 |
| rs10749053 | T/C | 0.0003 | 0.0033 | 0.89 | 0.0014 | 0.0014 | 0.33 | 0.0001 | 0.0010 | 0.87 |
| rs10741807 | T/C | 0.0006 | 0.0027 | 0.81 | 0.0009 | 0.0011 | 0.43 | 0.0011 | 0.0009 | 0.23 |
| rs4935786 | T/A | -0.0030 | 0.0026 | 0.29 | 0.0024 | 0.0011 | 0.04 | 0.0004 | 0.0008 | 0.67 |
| rs76097649 | A/G | 0.0038 | 0.0039 | 0.35 | -0.0010 | 0.0017 | 0.58 | 0.0000 | 0.0013 | 0.99 |
| rs4963776 | G/T | -0.0058 | 0.0030 | 0.05 | 0.0026 | 0.0013 | 0.03 | 0.0001 | 0.0009 | 0.93 |
| rs17380837 | C/T | 0.0010 | 0.0025 | 0.69 | -0.0012 | 0.0011 | 0.25 | -0.0013 | 0.0008 | 0.09 |
| rs12809354 | C/T | 0.0044 | 0.0032 | 0.17 | -0.0025 | 0.0014 | 0.07 | -0.0005 | 0.0010 | 0.59 |
| rs2860482 | A/C | -0.0020 | 0.0025 | 0.43 | 0.0023 | 0.0011 | 0.04 | 0.0015 | 0.0008 | 0.07 |
| rs71454237 | G/A | 0.0106 | 0.0028 | 0.00 | -0.0040 | 0.0012 | 9.3E-4 | 0.0010 | 0.0009 | 0.30 |
| rs12426679 | C/T | 0.0023 | 0.0023 | 0.31 | 0.0001 | 0.0010 | 0.92 | 0.0004 | 0.0007 | 0.57 |
| rs883079 | T/C | 0.0008 | 0.0026 | 0.78 | 0.0001 | 0.0011 | 0.91 | -0.0007 | 0.0008 | 0.36 |
| rs10773657 | C/A | -0.0049 | 0.0037 | 0.20 | 0.0022 | 0.0016 | 0.15 | -0.0004 | 0.0012 | 0.76 |
| rs6560886 | C/T | -0.0009 | 0.0029 | 0.76 | 0.0001 | 0.0012 | 0.86 | -0.0005 | 0.0009 | 0.57 |
| rs9506925 | T/C | 0.0001 | 0.0025 | 0.92 | -0.0010 | 0.0011 | 0.34 | -0.0004 | 0.0008 | 0.65 |
| rs35569628 | T/C | 0.0075 | 0.0027 | 0.01 | -0.0018 | 0.0011 | 0.11 | 0.0017 | 0.0009 | 0.05 |
| rs422068 | C/T | -0.0007 | 0.0024 | 0.70 | -0.0004 | 0.0010 | 0.70 | -0.0007 | 0.0008 | 0.33 |
| rs11156751 | C/T | 0.0001 | 0.0026 | 0.95 | -0.0013 | 0.0011 | 0.25 | -0.0005 | 0.0008 | 0.53 |
| rs73241997 | T/C | 0.0022 | 0.0032 | 0.50 | -0.0021 | 0.0014 | 0.12 | -0.0010 | 0.0010 | 0.30 |
| rs2738413 | A/G | 0.0006 | 0.0023 | 0.85 | 0.0003 | 0.0010 | 0.72 | -0.0002 | 0.0007 | 0.78 |
| rs74884082 | C/T | 0.0015 | 0.0027 | 0.57 | -0.0003 | 0.0011 | 0.78 | 0.0008 | 0.0009 | 0.34 |
| rs10873298 | C/T | -0.0009 | 0.0023 | 0.68 | 0.0000 | 0.0010 | 0.99 | 0.0004 | 0.0008 | 0.62 |
| rs147301839 | C/A | 0.0153 | 0.0183 | 0.42 | -0.0073 | 0.0077 | 0.39 | 0.0093 | 0.0059 | 0.12 |
| rs7170477 | A/G | 0.0030 | 0.0025 | 0.24 | -0.0012 | 0.0011 | 0.27 | 0.0007 | 0.0008 | 0.41 |
| rs74022964 | T/C | -0.0006 | 0.0031 | 0.91 | 0.0011 | 0.0013 | 0.43 | 0.0016 | 0.0010 | 0.11 |
| rs12908004 | G/A | 0.0013 | 0.0031 | 0.65 | 0.0001 | 0.0013 | 0.97 | 0.0001 | 0.0010 | 0.93 |
| rs4965430 | C/G | -0.0016 | 0.0024 | 0.52 | 0.0012 | 0.0010 | 0.23 | 0.0005 | 0.0008 | 0.53 |
| rs140185678 | A/G | 0.0029 | 0.0061 | 0.61 | -0.0012 | 0.0026 | 0.65 | -0.0003 | 0.0020 | 0.90 |
| rs2359171 | A/T | 0.0010 | 0.0030 | 0.76 | -0.0012 | 0.0013 | 0.36 | -0.0003 | 0.0010 | 0.73 |
| rs7225165 | G/A | 0.0018 | 0.0036 | 0.58 | -0.0006 | 0.0015 | 0.66 | -0.0004 | 0.0011 | 0.78 |
| rs9899183 | T/C | -0.0016 | 0.0025 | 0.52 | 0.0008 | 0.0011 | 0.47 | 0.0015 | 0.0008 | 0.07 |
| rs72811294 | G/C | -0.0051 | 0.0036 | 0.15 | -0.0009 | 0.0015 | 0.60 | -0.0022 | 0.0012 | 0.06 |
| rs11658278 | T/C | 0.0031 | 0.0023 | 0.16 | -0.0007 | 0.0010 | 0.48 | 0.0005 | 0.0007 | 0.50 |
| rs1563304 | T/C | 0.0094 | 0.0030 | 0.00 | 0.0002 | 0.0013 | 0.88 | 0.0038 | 0.0009 | 0.00 |
| rs12604076 | T/C | -0.0009 | 0.0023 | 0.68 | 0.0008 | 0.0010 | 0.38 | 0.0009 | 0.0007 | 0.23 |
| rs9953366 | C/T | 0.0026 | 0.0025 | 0.35 | -0.0001 | 0.0010 | 0.99 | 0.0010 | 0.0008 | 0.20 |
| rs8088085 | A/C | 0.0017 | 0.0023 | 0.44 | -0.0001 | 0.0010 | 0.96 | 0.0002 | 0.0007 | 0.84 |
| rs2834618 | T/G | 0.0000 | 0.0038 | 0.99 | 0.0009 | 0.0016 | 0.55 | 0.0015 | 0.0012 | 0.21 |
| rs464901 | T/C | -0.0028 | 0.0024 | 0.29 | 0.0013 | 0.0010 | 0.18 | 0.0005 | 0.0008 | 0.53 |
| rs133902 | T/C | 0.0014 | 0.0023 | 0.49 | 0.0005 | 0.0010 | 0.65 | 0.0012 | 0.0007 | 0.10 |

SNP=single nucleotide polymorphism; E/A=effect/alternative alleles; Beta = per allele effect on SD units; SE = standard error; P value = p-value for the genetic association.

Table 16. Characteristics of SNPs for heart failure instrument from GWAS meta-analysis

| SNP | Chromosome position | Nearest Gene | Alleles  (E/A) | EAF | Beta | SE | P value |
| --- | --- | --- | --- | --- | --- | --- | --- |
| rs660240 | 1:109817838 | CELSR2 | C/T | 0.79 | 0.0583 | 0.0371 | 3.25E-10 |
| rs17042102 | 4:111668626 | PITX2 | A/G | 0.12 | 0.1133 | 0.0937 | 5.71E-20 |
| rs11745324 | 5:137012171 | KLHL3 | G/A | 0.77 | 0.0488 | 0.0295 | 2.35E-08 |
| rs4135240 | 6:36647680 | CDKN1A | T/C | 0.66 | 0.0488 | 0.0295 | 6.84E-09 |
| rs55730499 | 6:161005610 | LPA | T/C | 0.07 | 0.1044 | 0.0830 | 1.83E-11 |
| rs140570886 | 6:161013013 | LPA | C/T | 0.02 | 0.2151 | 0.2727 | 7.69E-11 |
| rs1556516 | 9:22100176 | 9p21 | C/G | 0.48 | 0.0583 | 0.0371 | 1.57E-15 |
| rs600038 | 9:136151806 | ABO | C/T | 0.21 | 0.0583 | 0.0371 | 3.68E-09 |
| rs4746140 | 10:75417249 | SYNPO2L | G/C | 0.85 | 0.0677 | 0.0453 | 1.10E-09 |
| rs17617337 | 10:121426884 | BAG3 | C/T | 0.78 | 0.0583 | 0.0371 | 3.65E-09 |
| rs4766578 | 12:111904371 | ATXN2 | T/A | 0.47 | 0.0392 | 0.0223 | 4.90E-08 |
| rs56094641 | 16:53806453 | FTO | G/A | 0.42 | 0.0488 | 0.0295 | 1.21E-08 |

SNP=single nucleotide polymorphism; E/A=effect/alternative alleles; EAF=effect allele frequency; Beta = per allele effect on SD units; SE = standard error; P value = p-value for the genetic association.

Table 17. The raw information of heart failure SNPs with sleep duration

| SNP | Alleles  (E/A) | Continuous Sleep Duration | | | Short sleep duration | | | Long sleep duration | | |
| --- | --- | --- | --- | --- | --- | --- | --- | --- | --- | --- |
|  |  | Beta | SE | P value | Beta | SE | P value | Beta | SE | P value |
| rs660240 | C/T | -0.0013 | 0.0028 | 0.64 | -0.0012 | 0.0012 | 0.31 | -0.0009 | 0.0009 | 0.49 |
| rs17042102 | A/G | -0.0031 | 0.0038 | 0.41 | 0.0013 | 0.0016 | 0.40 | -0.0008 | 0.0012 | 0.11 |
| rs11745324 | G/A | -0.0028 | 0.0027 | 0.27 | 0.0002 | 0.0011 | 0.88 | -0.0013 | 0.0009 | 0.35 |
| rs4135240 | T/C | 0.0022 | 0.0024 | 0.35 | -0.0023 | 0.0010 | 0.02 | -0.0008 | 0.0008 | 0.75 |
| rs55730499 | T/C | -0.0018 | 0.0042 | 0.69 | 0.0004 | 0.0018 | 0.86 | -0.0006 | 0.0013 | 0.69 |
| rs140570886 | C/T | -0.0098 | 0.0093 | 0.28 | -0.0035 | 0.0039 | 0.38 | -0.0083 | 0.0030 | 0.42 |
| rs1556516 | C/G | -0.0005 | 0.0023 | 0.87 | -0.0008 | 0.0010 | 0.38 | -0.0011 | 0.0007 | 0.51 |
| rs600038 | C/T | 0.0048 | 0.0028 | 0.07 | -0.0019 | 0.0012 | 0.09 | -0.0009 | 0.0009 | 0.17 |
| rs4746140 | G/C | -0.0041 | 0.0032 | 0.22 | -0.0011 | 0.0014 | 0.39 | -0.0020 | 0.0010 | 0.06 |
| rs17617337 | C/T | 0.0006 | 0.0028 | 0.84 | 0.0011 | 0.0012 | 0.39 | 0.0003 | 0.0009 | 0.16 |
| rs4766578 | T/A | -0.0011 | 0.0023 | 0.62 | 0.0009 | 0.0010 | 0.33 | 0.0001 | 0.0007 | 0.99 |
| rs56094641 | G/A | 0.0162 | 0.0023 | 2.1E-12 | -0.0031 | 0.0010 | 1.4E-3 | 0.0037 | 0.0007 | 0.45 |

SNP=single nucleotide polymorphism; E/A=effect/alternative alleles; Beta = per allele effect on SD units; SE = standard error; P value = p-value for the genetic association.

Table 18. MR analysis for the association between sleep duration and AF and HF

| Outcome | Method | ***Atrial Fibrillation*** | |  | ***Heart Failure*** | |
| --- | --- | --- | --- | --- | --- | --- |
|  |  | Estimate/OR (95% CI) | P value |  | Estimate/OR (95% CI) | P value |
| ***Continuous Sleep Duration*** | Main IVW MR | -0.0038 (-0.0106-0.0031) | 0.280 |  | 0.0015 (-0.0248-0.0278) | 0.912 |
|  | Weighted median | 0.0002 (-0.0134-0.0138) | 0.977 |  | 0.0150 (-0.0265-0.0565) | 0.480 |
|  | MR-Egger | 0.0037 (-0.0163-0.0238) | 0.716 |  | 0.0498 (-0.0231-0.1226) | 0.181 |
| ***Short Sleep Duration*** | Main IVW MR | 1.002 (0.999-1.005) | 0.116 |  | 1.008 (0.997-1.019) | 0.153 |
|  | Weighted median | 1.002 (0.997-1.008) | 0.437 |  | 1.013 (0.994-1.032) | 0.178 |
|  | MR-Egger | 1.000 (0.993-1.007) | 0.923 |  | 0.991 (0.949-1.035) | 0.688 |
| ***Long Sleep Duration*** | Main IVW MR | 1.000 (0.998-1.002) | 0.903 |  | 1.006 (0.997-1.014) | 0.179 |
|  | Weighted median | 1.001 (0.997-1.006) | 0.592 |  | 1.007 (0.992-1.022) | 0.374 |
|  | MR-Egger | 1.003 (0.997-1.008) | 0.337 |  | 1.028 (0.998-1.059) | 0.064 |

OR = odds ratio; CI = confidence interval; P value is for the genetic association.

Table 19. The raw information of continuous sleep duration instrument SNPs with stroke

| SNP | Alleles  (E/A) | IS | | | CES | | | LAS | | | SVS | | |
| --- | --- | --- | --- | --- | --- | --- | --- | --- | --- | --- | --- | --- | --- |
|  |  | Beta | SE | P value | Beta | SE | P value | Beta | SE | P value | Beta | SE | P value |
| rs915416 | C/G | 0.0137 | 0.0110 | 0.213 | 0.0394 | 0.0212 | 0.063 | 0.0682 | 0.0272 | 0.012 | 0.0234 | 0.0256 | 0.360 |
| rs269054 | A/T | -0.0005 | 0.0105 | 0.960 | 0.0258 | 0.0200 | 0.197 | -0.0179 | 0.0260 | 0.490 | 0.0253 | 0.0243 | 0.297 |
| rs61796569 | T/C | -0.0013 | 0.0113 | 0.905 | 0.0074 | 0.0216 | 0.734 | 0.0056 | 0.0279 | 0.840 | 0.0191 | 0.0257 | 0.457 |
| rs12567114 | A/G | 0.0061 | 0.0113 | 0.588 | 0.0177 | 0.0220 | 0.421 | -0.0128 | 0.0281 | 0.649 | 0.0328 | 0.0261 | 0.208 |
| rs11190970 | G/A | -0.0002 | 0.0126 | 0.986 | 0.0329 | 0.0239 | 0.170 | 0.0036 | 0.0311 | 0.907 | -0.0363 | 0.0284 | 0.201 |
| rs7915425 | T/C | 0.0278 | 0.0131 | 0.034 | -0.0039 | 0.0254 | 0.877 | 0.0261 | 0.0320 | 0.415 | 0.0216 | 0.0303 | 0.477 |
| rs12246842 | A/G | 0.0090 | 0.0100 | 0.369 | 0.0149 | 0.0194 | 0.441 | 0.0046 | 0.0250 | 0.853 | 0.0068 | 0.0233 | 0.771 |
| rs10761674 | C/T | 0.0004 | 0.0098 | 0.965 | -0.0490 | 0.0188 | 0.009 | -0.0004 | 0.0243 | 0.988 | 0.0171 | 0.0226 | 0.447 |
| rs1939455 | G/T | 0.0185 | 0.0165 | 0.262 | -0.0320 | 0.0312 | 0.306 | 0.0350 | 0.0414 | 0.398 | -0.0008 | 0.0380 | 0.983 |
| rs7115226 | A/C | -0.0248 | 0.0203 | 0.222 | -0.0498 | 0.0407 | 0.221 | -0.0455 | 0.0519 | 0.381 | -0.0005 | 0.0484 | 0.992 |
| rs1263056 | A/G | -0.0048 | 0.0102 | 0.639 | 0.0185 | 0.0195 | 0.343 | -0.0205 | 0.0255 | 0.422 | -0.0253 | 0.0239 | 0.291 |
| rs7951019 | G/T | 0.0352 | 0.0348 | 0.312 | 0.0978 | 0.0738 | 0.185 | -0.0048 | 0.0935 | 0.959 | 0.1324 | 0.0788 | 0.093 |
| rs1057703 | G/T | -0.0010 | 0.0144 | 0.944 | 0.0475 | 0.0272 | 0.081 | 0.0302 | 0.0358 | 0.400 | 0.0235 | 0.0334 | 0.482 |
| rs1517572 | C/A | -0.0024 | 0.0102 | 0.813 | -0.0131 | 0.0196 | 0.504 | -0.0054 | 0.0253 | 0.832 | 0.0130 | 0.0235 | 0.579 |
| rs4592416 | G/A | -0.0283 | 0.0099 | 0.004 | -0.0011 | 0.0188 | 0.954 | -0.0628 | 0.0246 | 0.011 | -0.0297 | 0.0228 | 0.191 |
| rs11602180 | C/T | -0.0009 | 0.0139 | 0.948 | 0.0224 | 0.0269 | 0.405 | -0.0744 | 0.0335 | 0.027 | 0.0239 | 0.0319 | 0.455 |
| rs174560 | C/T | -0.0276 | 0.0111 | 0.013 | -0.0516 | 0.0218 | 0.018 | -0.0792 | 0.0281 | 0.005 | 0.0234 | 0.0258 | 0.364 |
| rs12791153 | T/A | -0.0045 | 0.0191 | 0.816 | 0.0309 | 0.0373 | 0.406 | -0.0622 | 0.0497 | 0.211 | 0.0146 | 0.0474 | 0.757 |
| rs1553132 | G/A | -0.0136 | 0.0113 | 0.230 | 0.0019 | 0.0220 | 0.932 | -0.0035 | 0.0282 | 0.902 | -0.0248 | 0.0264 | 0.349 |
| rs11614986 | A/G | 0.0006 | 0.0129 | 0.960 | 0.0072 | 0.0251 | 0.774 | -0.0110 | 0.0317 | 0.728 | 0.0108 | 0.0295 | 0.714 |
| rs4767550 | G/A | 0.0040 | 0.0103 | 0.697 | 0.0001 | 0.0195 | 0.997 | 0.0604 | 0.0256 | 0.018 | -0.0182 | 0.0239 | 0.447 |
| rs34354917 | C/A | -0.0087 | 0.0120 | 0.469 | -0.0552 | 0.0226 | 0.015 | -0.0031 | 0.0295 | 0.917 | 0.0155 | 0.0274 | 0.571 |
| rs6575005 | T/C | 0.0127 | 0.0129 | 0.326 | 0.0023 | 0.0227 | 0.920 | 0.0444 | 0.0296 | 0.134 | 0.0130 | 0.0275 | 0.636 |
| rs10483350 | G/A | 0.0295 | 0.0127 | 0.020 | 0.0310 | 0.0247 | 0.208 | 0.0100 | 0.0313 | 0.750 | 0.0420 | 0.0287 | 0.143 |
| rs61985058 | T/C | 0.0143 | 0.0152 | 0.347 | 0.0335 | 0.0334 | 0.316 | -0.0066 | 0.0377 | 0.862 | -0.0228 | 0.0356 | 0.522 |
| rs55658675 | C/T | 0.0039 | 0.0105 | 0.709 | 0.0141 | 0.0201 | 0.481 | 0.0145 | 0.0260 | 0.578 | -0.0262 | 0.0241 | 0.277 |
| rs11621908 | C/T | -0.0038 | 0.0185 | 0.839 | -0.0071 | 0.0358 | 0.843 | 0.0219 | 0.0455 | 0.631 | 0.0255 | 0.0433 | 0.555 |
| rs8038326 | A/G | -0.0056 | 0.0110 | 0.610 | -0.0021 | 0.0211 | 0.921 | 0.0114 | 0.0271 | 0.672 | -0.0547 | 0.0253 | 0.031 |
| rs11643715 | G/C | 0.0044 | 0.0110 | 0.689 | 0.0117 | 0.0212 | 0.579 | 0.0067 | 0.0273 | 0.805 | 0.0648 | 0.0256 | 0.011 |
| rs9940646 | C/G | -0.0111 | 0.0100 | 0.268 | -0.0084 | 0.0194 | 0.663 | -0.0494 | 0.0251 | 0.049 | -0.0196 | 0.0232 | 0.398 |
| rs8050478 | G/A | -0.0001 | 0.0100 | 0.993 | 0.0131 | 0.0193 | 0.495 | 0.0035 | 0.0251 | 0.889 | -0.0396 | 0.0234 | 0.090 |
| rs3095508 | C/A | 0.0120 | 0.0103 | 0.246 | 0.0006 | 0.0196 | 0.976 | 0.0330 | 0.0253 | 0.192 | -0.0122 | 0.0250 | 0.626 |
| rs205024 | T/C | -0.0104 | 0.0104 | 0.316 | -0.0069 | 0.0198 | 0.726 | -0.0678 | 0.0259 | 0.009 | 0.0045 | 0.0242 | 0.851 |
| rs2139261 | G/C | NA | NA | NA | NA | NA | NA | NA | NA | NA | NA | NA | NA |
| rs1991556 | G/A | NA | NA | NA | NA | NA | NA | NA | NA | NA | NA | NA | NA |
| rs9903973 | C/T | -0.0314 | 0.0100 | 0.002 | -0.0264 | 0.0190 | 0.165 | -0.0011 | 0.0246 | 0.964 | -0.0203 | 0.0231 | 0.380 |
| rs7503199 | C/T | -0.0021 | 0.0118 | 0.857 | -0.0101 | 0.0217 | 0.642 | 0.0016 | 0.0279 | 0.953 | -0.0092 | 0.0262 | 0.726 |
| rs12607679 | T/C | 0.0100 | 0.0115 | 0.386 | 0.0039 | 0.0220 | 0.861 | -0.0017 | 0.0281 | 0.950 | -0.0127 | 0.0259 | 0.625 |
| rs10421649 | A/T | 0.0093 | 0.0106 | 0.379 | 0.0206 | 0.0203 | 0.312 | -0.0023 | 0.0265 | 0.930 | -0.0061 | 0.0246 | 0.805 |
| rs7556815 | A/G | -0.0010 | 0.0122 | 0.935 | 0.0261 | 0.0235 | 0.266 | -0.0027 | 0.0306 | 0.930 | -0.0067 | 0.0286 | 0.814 |
| rs12611523 | A/G | 0.0052 | 0.0100 | 0.606 | -0.0098 | 0.0192 | 0.607 | 0.0446 | 0.0251 | 0.076 | -0.0059 | 0.0229 | 0.798 |
| rs4128364 | C/T | -0.0335 | 0.0106 | 0.001 | -0.0082 | 0.0202 | 0.684 | -0.0011 | 0.0259 | 0.967 | -0.0426 | 0.0242 | 0.078 |
| rs4538155 | T/C | 0.0207 | 0.0104 | 0.046 | 0.0245 | 0.0196 | 0.211 | -0.0190 | 0.0256 | 0.459 | 0.0292 | 0.0239 | 0.222 |
| rs11885663 | T/C | 0.0182 | 0.0114 | 0.111 | -0.0092 | 0.0219 | 0.674 | 0.0480 | 0.0277 | 0.084 | 0.0038 | 0.0259 | 0.884 |
| rs10173260 | C/T | 0.0021 | 0.0101 | 0.833 | -0.0159 | 0.0191 | 0.407 | 0.0312 | 0.0252 | 0.215 | -0.0307 | 0.0235 | 0.191 |
| rs374153 | C/T | 0.0043 | 0.0148 | 0.769 | 0.0106 | 0.0285 | 0.710 | 0.0011 | 0.0369 | 0.977 | -0.0198 | 0.0327 | 0.545 |
| rs75539574 | C/A | -0.0220 | 0.0182 | 0.227 | -0.0652 | 0.0360 | 0.070 | -0.0749 | 0.0465 | 0.107 | 0.0141 | 0.0424 | 0.740 |
| rs72804080 | G/A | -0.0014 | 0.0142 | 0.920 | -0.0051 | 0.0274 | 0.852 | 0.0266 | 0.0349 | 0.446 | 0.0342 | 0.0331 | 0.302 |
| rs62120041 | T/C | -0.0206 | 0.0210 | 0.325 | 0.0150 | 0.0423 | 0.724 | -0.0698 | 0.0519 | 0.179 | -0.0716 | 0.0488 | 0.142 |
| rs2072727 | T/C | 0.0001 | 0.0099 | 0.989 | -0.0278 | 0.0191 | 0.145 | -0.0437 | 0.0248 | 0.078 | -0.0263 | 0.0230 | 0.253 |
| rs7644809 | T/C | 0.0006 | 0.0101 | 0.954 | 0.0244 | 0.0193 | 0.206 | 0.0096 | 0.0254 | 0.705 | -0.0431 | 0.0238 | 0.070 |
| rs13088093 | G/T | -0.0206 | 0.0105 | 0.050 | -0.0110 | 0.0197 | 0.577 | -0.0300 | 0.0260 | 0.248 | -0.0377 | 0.0240 | 0.115 |
| rs7616632 | T/G | -0.0040 | 0.0100 | 0.689 | -0.0258 | 0.0189 | 0.172 | -0.0242 | 0.0246 | 0.325 | 0.0153 | 0.0230 | 0.506 |
| rs112230981 | A/G | -0.0551 | 0.0257 | 0.032 | -0.0841 | 0.0550 | 0.126 | -0.0253 | 0.0677 | 0.708 | -0.0701 | 0.0603 | 0.245 |
| rs17732997 | C/G | -0.0007 | 0.0101 | 0.942 | 0.0162 | 0.0194 | 0.403 | 0.0021 | 0.0247 | 0.933 | 0.0131 | 0.0232 | 0.573 |
| rs13109404 | T/G | -0.0019 | 0.0233 | 0.937 | 0.0449 | 0.0467 | 0.337 | -0.0538 | 0.0553 | 0.331 | -0.0194 | 0.0524 | 0.712 |
| rs2192528 | A/G | -0.0033 | 0.0101 | 0.743 | -0.0120 | 0.0193 | 0.535 | -0.0219 | 0.0252 | 0.385 | 0.0046 | 0.0234 | 0.843 |
| rs17427571 | A/G | 0.0059 | 0.0106 | 0.578 | -0.0071 | 0.0200 | 0.724 | -0.0129 | 0.0260 | 0.619 | 0.0347 | 0.0242 | 0.153 |
| rs35531607 | C/T | -0.0053 | 0.0099 | 0.592 | 0.0085 | 0.0190 | 0.655 | 0.0193 | 0.0245 | 0.431 | 0.0435 | 0.0228 | 0.057 |
| rs56372231 | T/C | -0.0003 | 0.0105 | 0.975 | 0.0240 | 0.0200 | 0.231 | -0.0188 | 0.0260 | 0.470 | -0.0200 | 0.0241 | 0.406 |
| rs180769 | T/C | 0.0090 | 0.0105 | 0.394 | 0.0315 | 0.0200 | 0.115 | 0.0262 | 0.0261 | 0.316 | 0.0005 | 0.0244 | 0.983 |
| rs11567976 | T/C | -0.0121 | 0.0100 | 0.225 | -0.0067 | 0.0190 | 0.723 | -0.0599 | 0.0246 | 0.015 | -0.0326 | 0.0228 | 0.152 |
| rs365663 | A/G | 0.0000 | 0.0102 | 0.997 | -0.0095 | 0.0194 | 0.625 | -0.0194 | 0.0251 | 0.440 | 0.0287 | 0.0235 | 0.222 |
| rs151014368 | A/G | -0.0071 | 0.0122 | 0.563 | -0.0080 | 0.0233 | 0.732 | -0.0337 | 0.0301 | 0.263 | -0.0053 | 0.0283 | 0.851 |
| rs460692 | C/T | 0.0064 | 0.0147 | 0.666 | 0.0448 | 0.0284 | 0.115 | -0.0105 | 0.0371 | 0.778 | 0.0028 | 0.0349 | 0.937 |
| rs34556183 | A/G | NA | NA | NA | NA | NA | NA | NA | NA | NA | NA | NA | NA |
| rs80193650 | G/A | 0.0053 | 0.0139 | 0.705 | -0.0065 | 0.0266 | 0.807 | -0.0060 | 0.0336 | 0.859 | 0.0305 | 0.0327 | 0.350 |
| rs113113059 | T/C | -0.0050 | 0.0123 | 0.684 | 0.0041 | 0.0232 | 0.859 | -0.0140 | 0.0301 | 0.642 | -0.0433 | 0.0278 | 0.120 |
| rs9382445 | T/C | 0.0055 | 0.0105 | 0.601 | 0.0156 | 0.0203 | 0.441 | -0.0136 | 0.0268 | 0.613 | -0.0131 | 0.0245 | 0.593 |
| rs2231265 | G/A | 0.0092 | 0.0119 | 0.441 | 0.0069 | 0.0228 | 0.761 | 0.0235 | 0.0293 | 0.423 | -0.0050 | 0.0273 | 0.854 |
| rs9345234 | C/A | 0.0057 | 0.0100 | 0.567 | 0.0225 | 0.0192 | 0.240 | 0.0470 | 0.0251 | 0.062 | -0.0331 | 0.0233 | 0.155 |
| rs2079070 | C/G | -0.0057 | 0.0115 | 0.618 | -0.0217 | 0.0221 | 0.327 | -0.0120 | 0.0281 | 0.670 | -0.0196 | 0.0263 | 0.456 |
| rs7806045 | T/C | -0.0135 | 0.0115 | 0.240 | -0.0117 | 0.0224 | 0.600 | -0.0112 | 0.0286 | 0.695 | -0.0320 | 0.0266 | 0.228 |
| rs34731055 | T/C | 0.0120 | 0.0103 | 0.246 | 0.0220 | 0.0245 | 0.369 | -0.0180 | 0.0317 | 0.570 | -0.0036 | 0.0294 | 0.903 |
| rs73219758 | G/A | 0.0014 | 0.0110 | 0.898 | 0.0143 | 0.0213 | 0.502 | -0.0363 | 0.0270 | 0.179 | 0.0046 | 0.0253 | 0.855 |
| rs330088 | C/T | -0.0079 | 0.0105 | 0.453 | -0.0396 | 0.0200 | 0.047 | 0.0180 | 0.0262 | 0.493 | -0.0178 | 0.0240 | 0.457 |
| rs1776776 | T/C | -0.0094 | 0.0148 | 0.527 | 0.0351 | 0.0295 | 0.235 | -0.0191 | 0.0371 | 0.607 | -0.0093 | 0.0347 | 0.788 |
| rs10973207 | T/G | 0.0067 | 0.0141 | 0.635 | -0.0334 | 0.0268 | 0.214 | 0.0310 | 0.0344 | 0.368 | 0.0709 | 0.0316 | 0.025 |

SNP=single nucleotide polymorphism; E/A=effect/alternative alleles; Beta = per allele effect on SD units; SE = standard error; P value = p-value for the genetic association.

Table 20. The raw information of long sleep duration instrument SNPs with stroke

| SNP | Alleles  (E/A) | IS | | | CES | | | LAS | | | SVS | | |
| --- | --- | --- | --- | --- | --- | --- | --- | --- | --- | --- | --- | --- | --- |
|  |  | Beta | SE | P value | Beta | SE | P value | Beta | SE | P value | Beta | SE | P value |
| rs6737318 | A/G | 0.0013 | 0.0121 | 0.917 | -0.0197 | 0.0233 | 0.397 | -0.0012 | 0.0302 | 0.968 | -0.0015 | 0.0282 | 0.958 |
| rs75458655 | C/T | -0.0047 | 0.0415 | 0.909 | -0.0397 | 0.0863 | 0.645 | -0.1666 | 0.1047 | 0.112 | 0.0217 | 0.0962 | 0.821 |
| rs17688916 | T/A | NA | NA | NA | NA | NA | NA | NA | NA | NA | NA | NA | NA |
| rs17817288 | A/G | -0.0110 | 0.0100 | 0.271 | -0.0013 | 0.0190 | 0.944 | -0.0201 | 0.0248 | 0.419 | -0.0141 | 0.0231 | 0.540 |
| rs549961083 | C/T | NA | NA | NA | NA | NA | NA | NA | NA | NA | NA | NA | NA |
| rs3751046 | A/G | 0.0018 | 0.0143 | 0.901 | -0.0539 | 0.0278 | 0.052 | -0.0300 | 0.0354 | 0.396 | -0.0173 | 0.0332 | 0.602 |
| rs7534398 | T/A | -0.0122 | 0.0126 | 0.332 | -0.0142 | 0.0244 | 0.560 | -0.0290 | 0.0308 | 0.346 | 0.0115 | 0.0287 | 0.689 |
| rs10899257 | G/A | 0.0078 | 0.0142 | 0.582 | -0.0124 | 0.0275 | 0.652 | -0.0038 | 0.0351 | 0.913 | -0.0357 | 0.0329 | 0.278 |

SNP=single nucleotide polymorphism; E/A=effect/alternative alleles; Beta = per allele effect on SD units; SE = standard error; P value = p-value for the genetic association.

Table 21. The raw information of short sleep duration instrument SNPs with stroke

| SNP | Alleles  (E/A) | IS | | | CES | | | LAS | | | SVS | | |
| --- | --- | --- | --- | --- | --- | --- | --- | --- | --- | --- | --- | --- | --- |
|  |  | Beta | SE | P value | Beta | SE | P value | Beta | SE | P value | Beta | SE | P value |
| rs2820313 | A/G | -0.0171 | 0.0105 | 0.104 | 0.0038 | 0.0201 | 0.851 | -0.0186 | 0.0260 | 0.476 | -0.0580 | 0.0241 | 0.016 |
| rs7524118 | T/C | 0.0151 | 0.0111 | 0.173 | 0.0323 | 0.0212 | 0.128 | 0.0698 | 0.0274 | 0.011 | 0.0291 | 0.0259 | 0.262 |
| rs2186122 | A/T | -0.0160 | 0.0100 | 0.111 | -0.0230 | 0.0191 | 0.228 | -0.0122 | 0.0248 | 0.621 | 0.0044 | 0.0230 | 0.847 |
| rs12567114 | G/A | -0.0061 | 0.0113 | 0.588 | -0.0177 | 0.0220 | 0.421 | 0.0128 | 0.0281 | 0.649 | -0.0328 | 0.0261 | 0.208 |
| rs1607227 | G/T | -0.0050 | 0.0111 | 0.654 | 0.0138 | 0.0216 | 0.523 | -0.0223 | 0.0279 | 0.424 | -0.0218 | 0.0258 | 0.398 |
| rs7939345 | T/G | 0.0020 | 0.0127 | 0.878 | -0.0265 | 0.0244 | 0.278 | 0.0462 | 0.0306 | 0.131 | -0.0522 | 0.0292 | 0.074 |
| rs17388803 | A/C | -0.0317 | 0.0157 | 0.044 | -0.0741 | 0.0298 | 0.013 | -0.0134 | 0.0391 | 0.732 | -0.0322 | 0.0366 | 0.378 |
| rs59779556 | T/G | -0.0014 | 0.0101 | 0.890 | -0.0177 | 0.0191 | 0.354 | -0.0115 | 0.0251 | 0.645 | 0.0309 | 0.0234 | 0.187 |
| rs205024 | C/T | 0.0104 | 0.0104 | 0.316 | 0.0069 | 0.0198 | 0.726 | 0.0678 | 0.0259 | 0.009 | -0.0045 | 0.0242 | 0.851 |
| rs12963463 | C/T | -0.0086 | 0.0111 | 0.436 | -0.0035 | 0.0212 | 0.869 | 0.0129 | 0.0272 | 0.637 | 0.0118 | 0.0248 | 0.634 |
| rs2863957 | C/A | 0.0027 | 0.0120 | 0.822 | -0.0197 | 0.0232 | 0.396 | 0.0042 | 0.0301 | 0.888 | 0.0005 | 0.0281 | 0.986 |
| rs1380703 | A/G | -0.0040 | 0.0106 | 0.709 | 0.0120 | 0.0203 | 0.555 | 0.0133 | 0.0260 | 0.610 | -0.0094 | 0.0245 | 0.702 |
| rs75539574 | A/C | 0.0220 | 0.0182 | 0.227 | 0.0652 | 0.0360 | 0.070 | 0.0749 | 0.0465 | 0.107 | -0.0141 | 0.0424 | 0.740 |
| rs5757675 | G/T | 0.0024 | 0.0114 | 0.836 | 0.0457 | 0.0212 | 0.031 | -0.0124 | 0.0275 | 0.650 | -0.0268 | 0.0259 | 0.301 |
| rs2014830 | C/T | 0.0119 | 0.0109 | 0.274 | -0.0305 | 0.0209 | 0.143 | 0.0236 | 0.0272 | 0.385 | 0.0095 | 0.0252 | 0.705 |
| rs13107325 | C/T | 0.0065 | 0.0215 | 0.761 | 0.0299 | 0.0435 | 0.492 | -0.0206 | 0.0529 | 0.697 | -0.0234 | 0.0475 | 0.622 |
| rs17005118 | G/A | -0.0120 | 0.0112 | 0.286 | -0.0221 | 0.0215 | 0.304 | -0.0275 | 0.0279 | 0.325 | 0.0256 | 0.0256 | 0.317 |
| rs3776864 | A/C | -0.0026 | 0.0110 | 0.814 | -0.0268 | 0.0211 | 0.204 | 0.0278 | 0.0273 | 0.309 | 0.0292 | 0.0253 | 0.249 |
| rs4585442 | G/A | -0.0034 | 0.0107 | 0.747 | -0.0220 | 0.0211 | 0.297 | 0.0275 | 0.0274 | 0.316 | -0.0134 | 0.0253 | 0.597 |
| rs12518468 | T/C | -0.0007 | 0.0109 | 0.953 | -0.0161 | 0.0209 | 0.441 | -0.0044 | 0.0270 | 0.870 | -0.0172 | 0.0253 | 0.496 |
| rs9321171 | C/T | 0.0013 | 0.0098 | 0.891 | -0.0122 | 0.0193 | 0.527 | 0.0259 | 0.0253 | 0.305 | -0.0151 | 0.0235 | 0.521 |
| rs142180737 | T/C | NA | NA | NA | NA | NA | NA | NA | NA | NA | NA | NA | NA |
| rs12661667 | C/T | 0.0145 | 0.0113 | 0.200 | 0.0275 | 0.0220 | 0.212 | 0.0154 | 0.0280 | 0.582 | 0.0022 | 0.0259 | 0.933 |
| rs9367621 | T/A | 0.0027 | 0.0100 | 0.789 | -0.0019 | 0.0192 | 0.921 | 0.0236 | 0.0249 | 0.345 | 0.0389 | 0.0231 | 0.093 |
| rs1229762 | C/T | 0.0005 | 0.0105 | 0.961 | -0.0128 | 0.0200 | 0.523 | -0.0276 | 0.0260 | 0.290 | 0.0099 | 0.0243 | 0.683 |
| rs11763750 | G/A | 0.0116 | 0.0127 | 0.360 | -0.0095 | 0.0244 | 0.697 | 0.0246 | 0.0314 | 0.433 | -0.0010 | 0.0292 | 0.973 |
| rs60882754 | A/T | 0.0092 | 0.0233 | 0.693 | -0.0317 | 0.0453 | 0.484 | 0.0720 | 0.0589 | 0.222 | -0.0410 | 0.0515 | 0.555 |

SNP=single nucleotide polymorphism; E/A=effect/alternative alleles; Beta = per allele effect on SD units; SE = standard error; P value = p-value for the genetic association.

Table 22. MR analysis for the association between sleep duration and stroke

| Outcome | Method | ***Continuous Sleep Duration*** | | ***Short Sleep Duration*** | | ***Long Sleep Duration*** | |
| --- | --- | --- | --- | --- | --- | --- | --- |
|  |  | OR  (95% CI) | P value | OR  (95% CI) | P value | OR  (95% CI) | P value |
| IS | Main IVW MR | 0.943  (0.793-1.123) | 0.512 | 1.103  (0.968-1.256) | 0.142 | 0.970  (0.820-1.148) | 0.725 |
|  | Weighted median | 0.978  (0.764-1.252) | 0.861 | 1.054  (0.879-1.266) | 0.569 | 0.979  (0.797-1.204) | 0.842 |
|  | MR-Egger | 1.024  (0.526-1.991) | 0.945 | 1.174  (0.691-1.994) | 0.553 | 1.064  (0.662-1.709) | 0.798 |
| CES | Main IVW MR | 1.106  (0.800-1.529) | 0.542 | 0.989  (0.743-1.316) | 0.938 | 1.351  (0.973-1.877) | 0.072 |
|  | Weighted median | 1.227  (0.766-1.964) | 0.395 | 0.751  (0.515-1.095) | 0.136 | 1.278  (0.848-1.925) | 0.241 |
|  | MR-Egger | 1.940  (0.559-6.733) | 0.296 | 1.239  (0.376-4.084) | 0.725 | 1.599  (0.620-4.126) | 0.331 |
| LAS | Main IVW MR | 0.741  (0.477-1.153) | 0.184 | 1.395  (1.001-1.946) | 0.050 | 1.254  (0.825-1.906) | 0.289 |
|  | Weighted median | 0.895  (0.478-1.675) | 0.728 | 1.421  (0.895-2.255) | 0.136 | 1.086  (0.639-1.848) | 0.760 |
|  | MR-Egger | 0.518  (0.094-2.841) | 0.449 | 1.803  (0.456-7.126) | 0.400 | 2.506  (0.765-8.207) | 0.129 |
| SVS | Main IVW MR | 0.855  (0.582-1.257) | 0.426 | 1.017  (0.753-1.375) | 0.911 | 1.056  (0.714-1.560) | 0.786 |
|  | Weighted median | 0.845  (0.471-1.517) | 0.572 | 0.986  (0.641-1.517) | 0.950 | 1.013  (0.625-1.644) | 0.957 |
|  | MR-Egger | 1.353  (0.309-5.920) | 0.688 | 0.663  (0.198-2.221) | 0.505 | 1.326  (0.443-3.976) | 0.614 |

IS, Ischemic stroke; CES, cardioembolic stroke; LAS, large artery stroke; SVS, small vessel stroke; OR = odds ratio; CI = confidence interval; P value is for the genetic association.

Figure 1. Scatter plots and forest plots for the causal effect of continuous sleep duration on AF


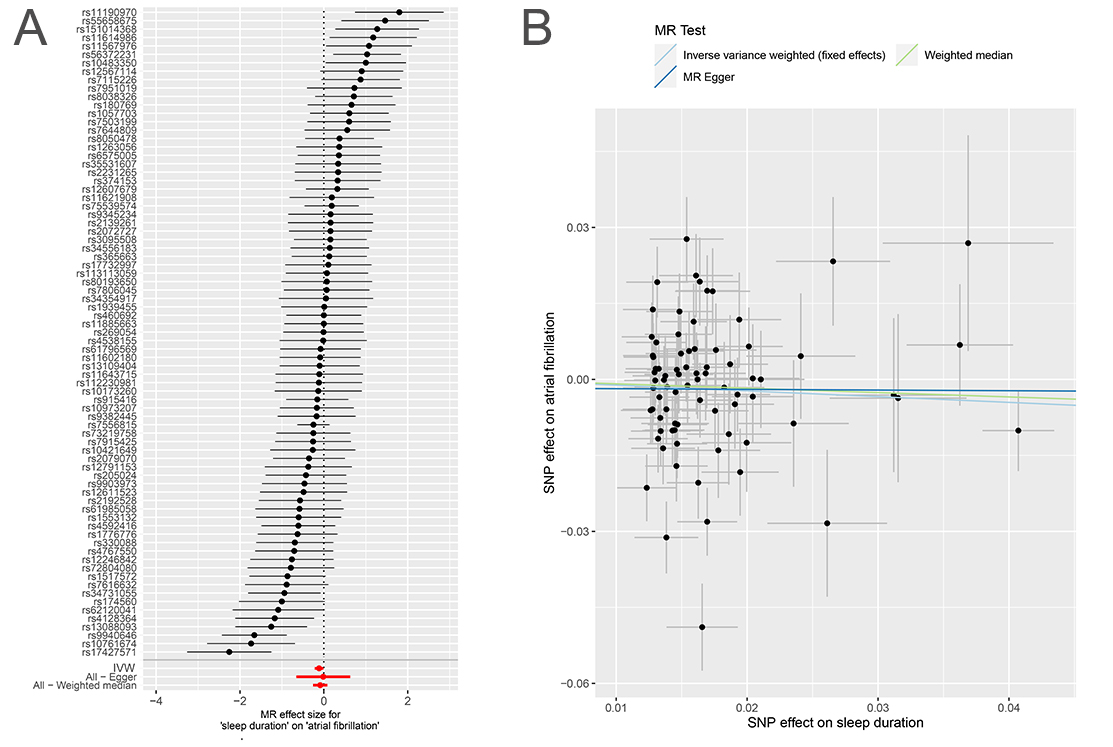


Figure 2. Scatter plots and forest plots for the causal effect of continuous sleep duration on HF


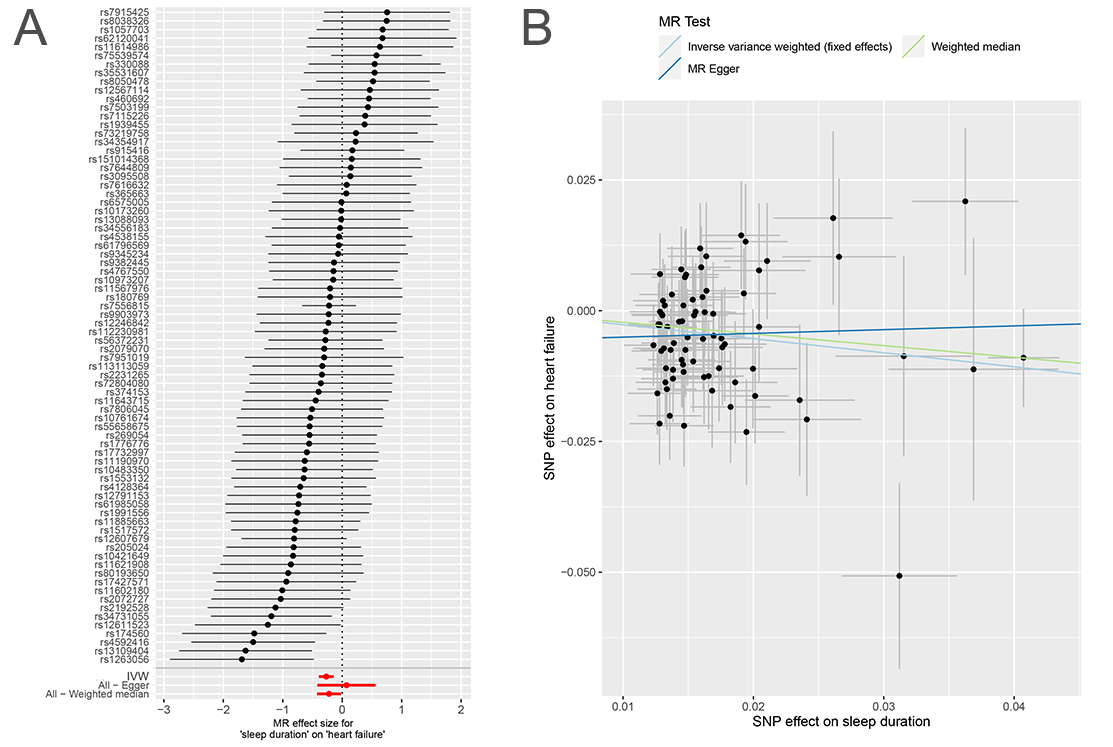


Figure 3. Scatter plots and forest plots for the causal effect of short sleep duration on AF and HF


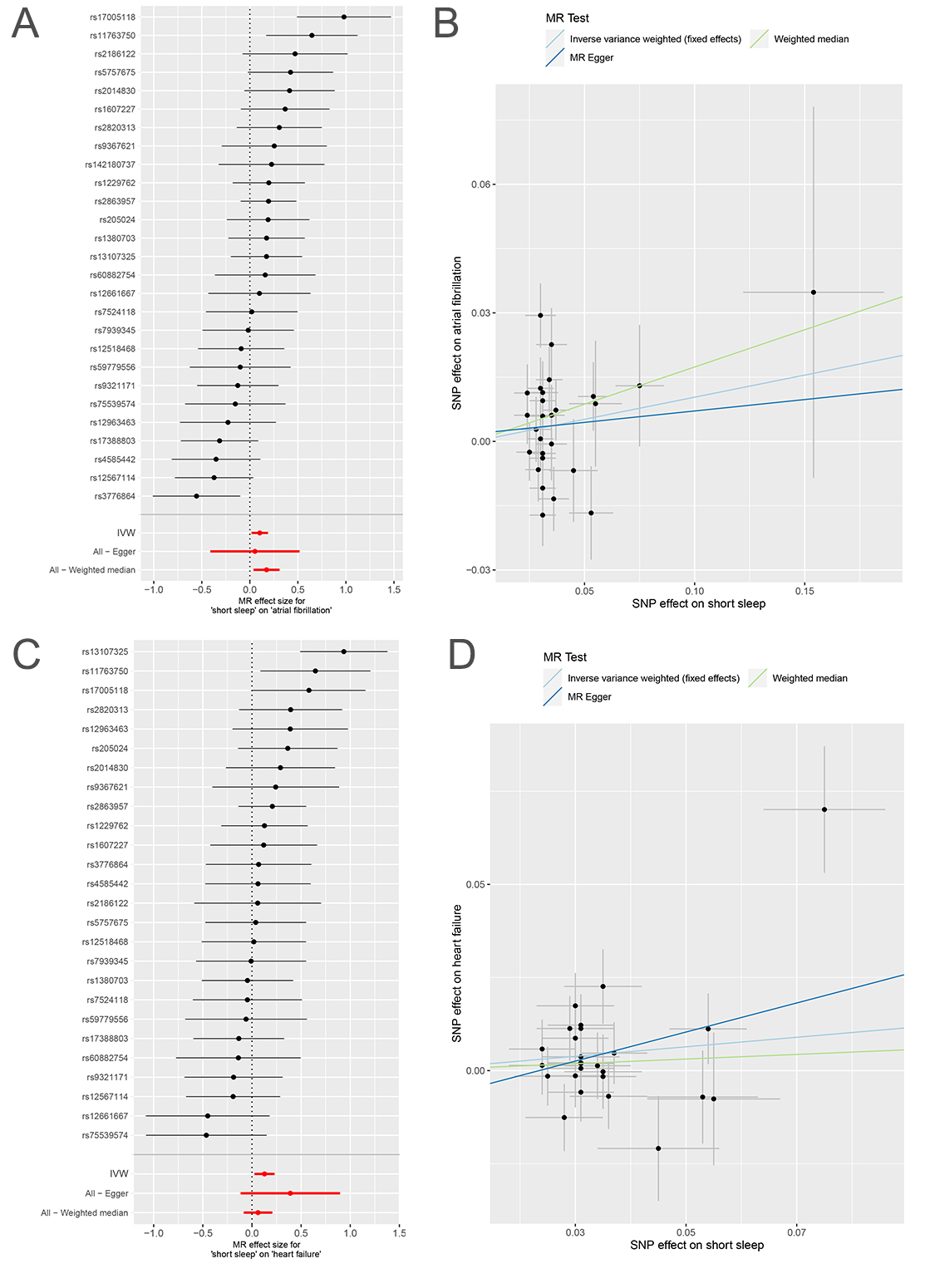


Figure 4. Scatter plots and forest plots for the causal effect of long sleep duration on AF and HF


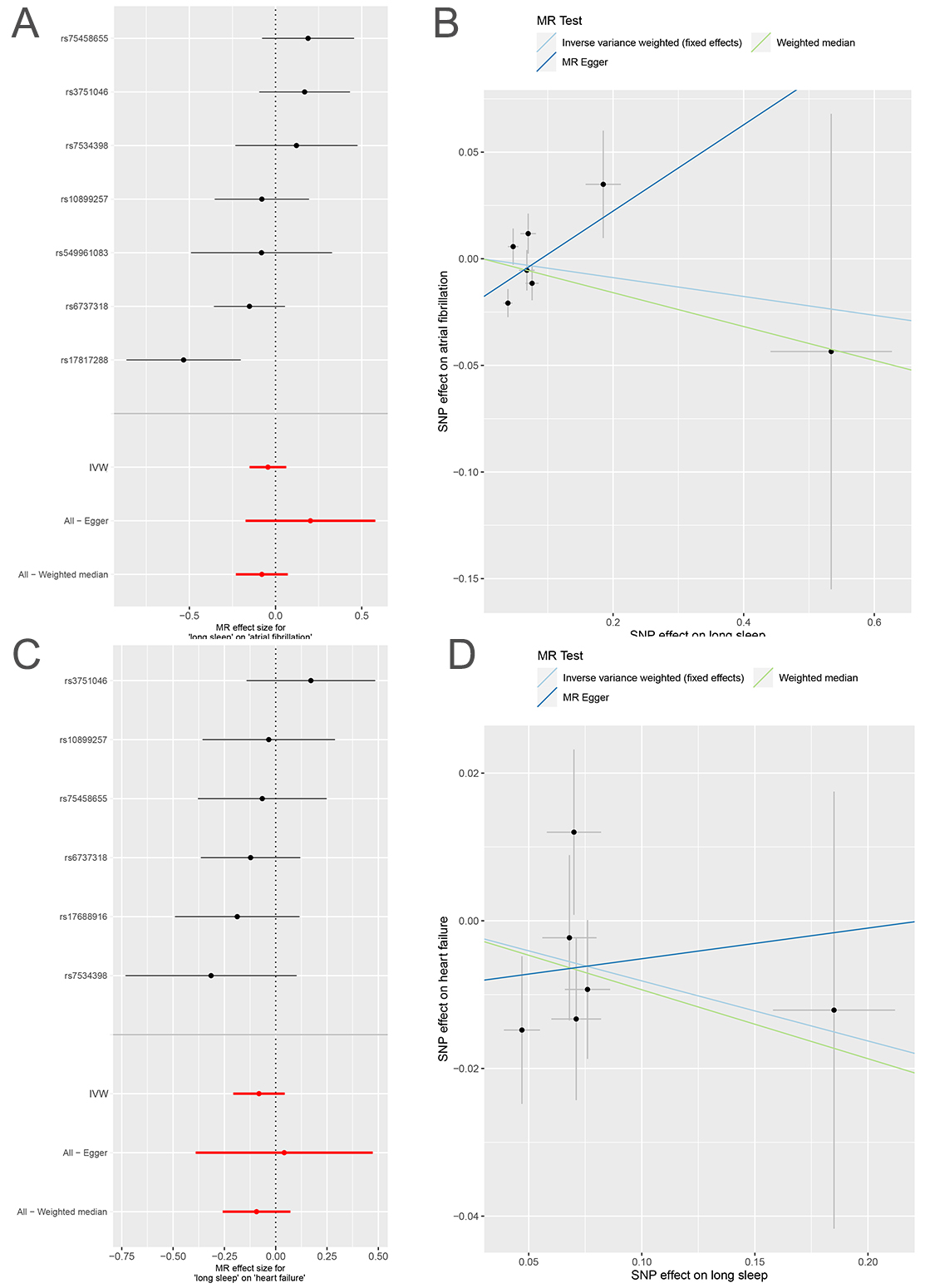


Figure 5. Cis-MR analysis for the association between sleep duration and AF and HF


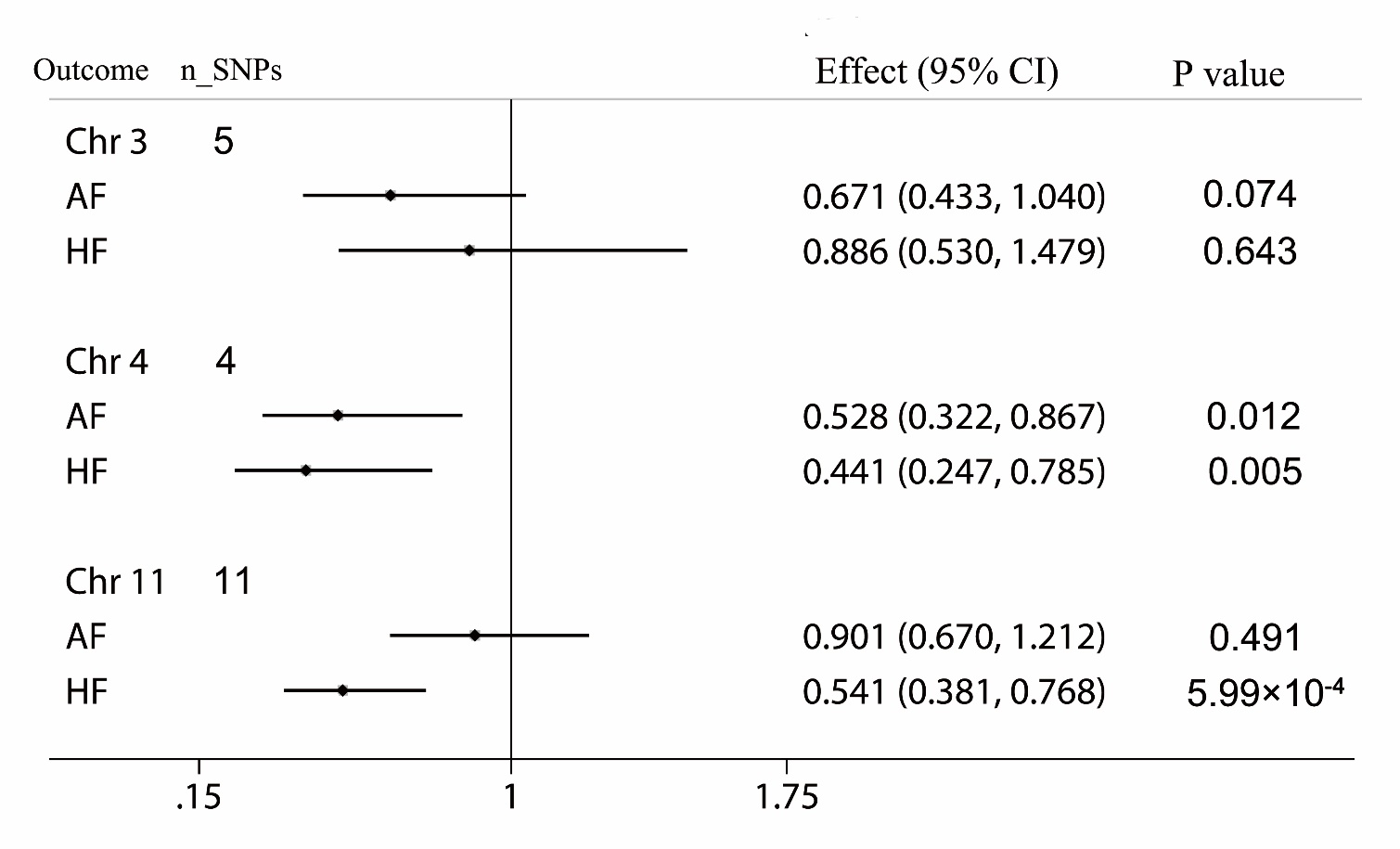


**Process of the MR**

Firstly, SNPs associated with sleep duration at genome-wide significance (P<5×10-8) were extracted from the original GWAS meta-analysis (Genome-wide association study identifies genetic loci for self-reported habitual sleep duration supported by accelerometer-derived estimates). In this step, 78 SNPs associated with continuous sleep duration, 27 SNPs associated with short sleep duration, and 8 SNPs associated with long sleep duration were preliminarily selected. Secondly, since the instrumental variables in MR study should be independent, we performed the linkage disequilibrium test on the LD-link website (https://ldlink.nci.nih.gov/) using LD-matrix tool based on Europeans (r2 < 0.1). Thirdly, genetic association data for AF and HF were extracted from two GWAS meta-analysis and we compared the effect allele of each SNP to harmonize the exposure and outcome data. Finally, all statistical analyses were performed using R software (version 3.6.1) with the Mendelian Randomization package, specifically as follows:


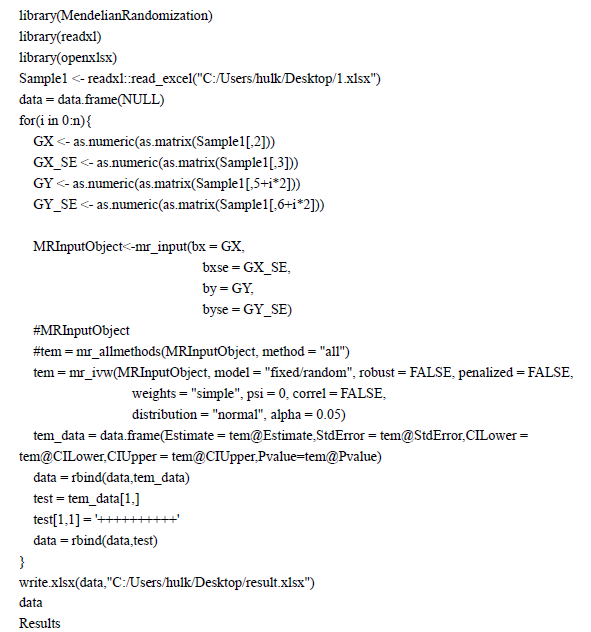

Supplement: Supplementary file 1 [file Table_1.docx]
